# Stress Induces Contextual Blindness in Lotteries and Coordination Games

**DOI:** 10.3389/fnbeh.2017.00236

**Published:** 2017-12-11

**Authors:** Isabelle Brocas, Juan D. Carrillo, Ryan Kendall

**Affiliations:** ^1^LABEL and Department of Economics, University of Southern California, Los Angeles, CA, United States; ^2^Department of Economics, University College London, London, United Kingdom

**Keywords:** stress, contextual blindness, lotteries, coordination games, risk taking

## Abstract

In this paper, we study how stress affects risk taking in three tasks: individual lotteries, Stag Hunt (coordination) games, and Hawk-Dove (anti-coordination) games. Both control and stressed subjects take more risks in all three tasks when the value of the safe option is decreased and in lotteries when the expected gain is increased. Also, subjects take longer to take decisions when stakes are high, when the safe option is less attractive and in the conceptually more difficult Hawk-Dove game. Stress (weakly) increases reaction times in those cases. Finally, our main result is that the behavior of stressed subjects in lotteries, Stag Hunt and Hawk-Dove are all highly predictive of each other (*p*-value < 0.001 for all three pairwise correlations). Such strong relationship is not present in our control group. Our results illustrate a “contextual blindness” caused by stress. The mathematical and behavioral tensions of Stag Hunt and Hawk-Dove games are axiomatically different, and we should expect different behavior across these games, and also with respect to the individual task. A possible explanation for the highly significant connection across tasks in the stress condition is that stressed subjects habitually rely on one mechanism to make a decision in all contexts whereas unstressed subjects utilize a more cognitively flexible approach.

## 1. Introduction

How does stress influence human behavior? While a significant amount of the work in this direction connects chronic stress with poor health outcomes, stress has also been shown to influence decision-making. The pioneering theory suggests that any stress above an optimal level unambiguously decreases performance (Yerkes-Dodson Law, Yerkes and Dodson, 1908). In spite of this Law's intuitive appeal, subsequent research has unveiled a far more subtle relationship between stress and choice, even in purely objective tasks^1^. In particular, the recent literature has shown a complex relationship between stress and an individual's preference to take risks (reviews in Mather and Lighthall, 2012; Starcke and Brand, 2012). Studies using incentivized lotteries find that stressed males choose more risky lotteries while stressed females choose less risky lotteries (Preston et al., 2007; Lighthall et al., 2009; Van Den Bos et al., 2009)^2^. In addition, compared to a one-time increase in stress, chronic stress experienced over the course of 8 days has been shown to more significantly increase risk-aversion (Kandasamy et al., 2014). Finally, cortisol has been shown to play a role in the preference of subjects to avoid ambiguity—a concept closely related to risk (Danese et al., 2017).

There is also a small literature studying the relationship between individual lotteries and two-player coordination (“Stag Hunt”) and anti-coordination (“Hawk-Dove”) strategic situations (or “games”). Results in this area are inconclusive. While some papers suggest a correlation between risk taking in individual lotteries and risk taking in Stag Hunt games (Heinemann et al., 2009; Chierchia and Coricelli, 2015), others do not find any significant relationship (Neumann and Vogt, 2009; Al-Ubaydli et al., 2013; Büyükboyacı, 2014). Imaging studies have found correlations in neural activity between choices in lotteries and Stag Hunt games but no correlation between choices in lotteries and Hawk-Dove games or between choices in the two games (Nagel et al., 2014). The authors conclude that Stag Hunt games engage brain networks associated to risk while Hawk-Dove games engage brain networks associated to strategic thinking.

**Figure 1:**
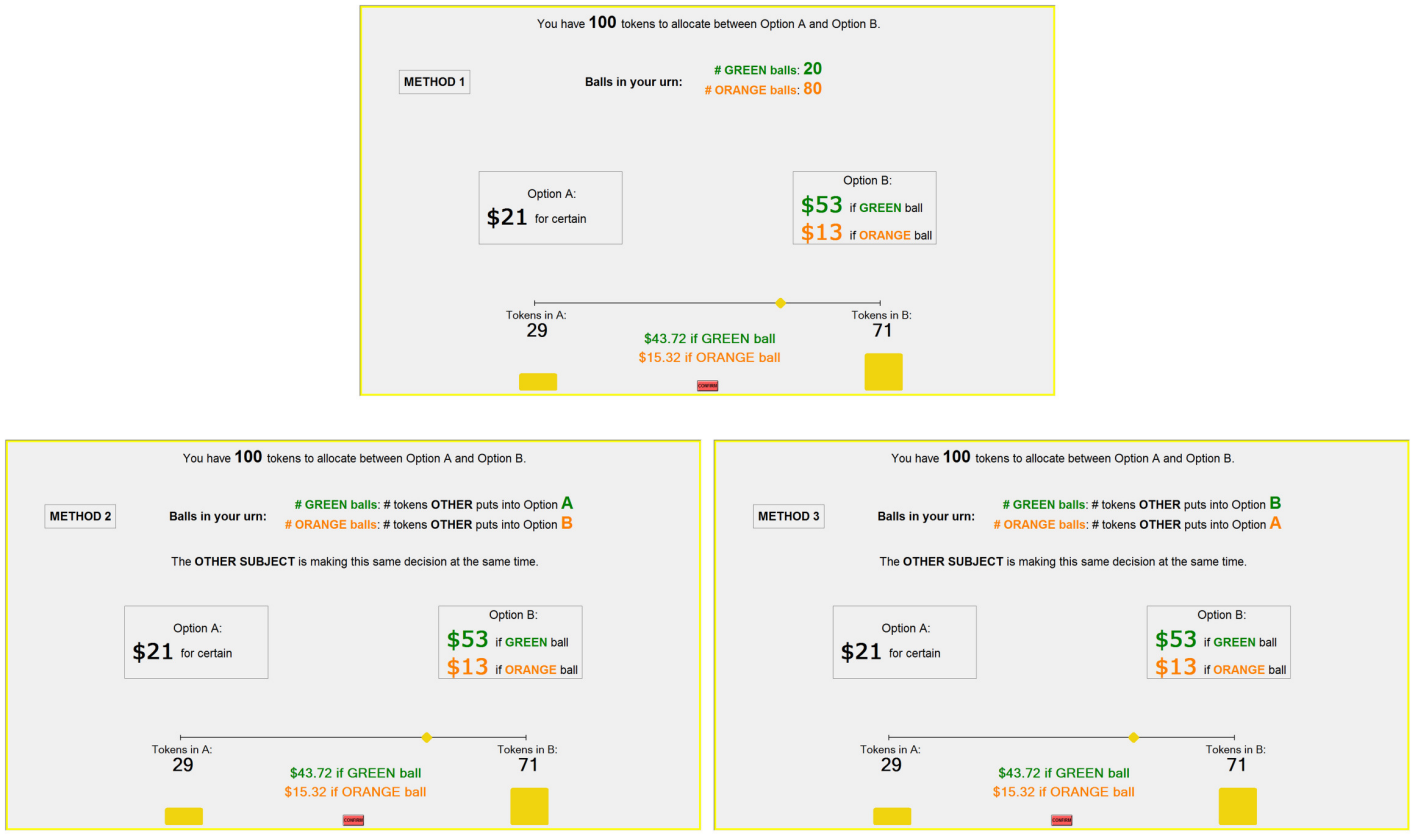
Screenshot of **LO** (Method 1), **HD** (Method 2), and **SH** (Method 3).

Our paper lies at the intersection of these two literatures by studying the effect of stress on risk-taking in lotteries and multi-player games of strategy—Stag Hunt and Hawk-Dove^3^. Our laboratory experiment relies on a novel way to represent these three tasks in an identical context that differs in the minimal amount to uniquely distinguish each task (Figure 1). Using this method, differences in behavior across tasks can best be explained by cognitive flexibility in response to fundamental differences across tasks rather than spurious differences in presentations.

Our first result is to show that subjects in both the control and stress condition behave in line with our theoretical predictions. In particular, our participants take more risks in all three tasks as the value of the safe option is decreased. They also take more risks in the individual lottery choice as the probability of the high payoff is increased (Result 1). Our second and main result is that stress impairs cognitive flexibility. More precisely, the choices made by stressed subjects in lotteries, Stag Hunt and Hawk-Dove are all highly and positively correlated with each other. In contrast, control subjects show a (weak) correlation between lotteries and Stag Hunt and no significant correlation between the other pairs of tasks. A cluster analysis reveals that about one-half of the subjects under stress allocate a similar and significant fraction of their endowment to the safe option in all tasks. These subjects are responsible for strengthening the behavioral relationship between tasks (Result 2). Finally, we show that subjects take more time to respond when stakes are high, when the safe option is less attractive and in Hawk-Dove (arguably, the conceptually more difficult game). Stress also tends to increase reaction times in all tasks (Result 3).

The findings suggest that some subjects under stress are oblivious to the fundamental differences that distinguish the three tasks (objective probabilities of lotteries, strategic complementarity of risk-taking in Stag Hunt, and strategic substitutability of risk-taking in Hawk-Dove). This *contextual blindness* fits in with recent findings which demonstrate that stress promotes habits in humans at the expense of goal-directed performance (Schwabe and Wolf, 2009). It has been shown that people under stress have an increased reliance on automatic over controlled cognitive processes (Schwabe et al., 2012) and are less likely to adjust their initial strategies (Kassam et al., 2009). One underlying mechanism that could lead to contextual blindness is the suppressed activation in the left temporoparietal junction (TPJ) caused by a stressful environment (Engelmann et al., 2017). Impairment of the TPJ has been shown to negatively impact a subject's ability to understand and predict the behavior of others (Samson et al., 2004) which is particularly important in games such as Hawk-Dove. Taken together, the results provide a framework for stress inducing intuitive, rather than deliberative, decision-making (Yu, 2016). Interestingly, previous research on decision-making under risk and stress has made it clear that “such habitual responses do not map neatly onto risk-aversion or risk-seeking” (Buchanan and Preston, 2014). Our paper shows that, rather than a story connecting stress and risk preferences, there is a more complex relationship between stress and risk evaluation across contexts.

A main implication of contextual blindness is that subjects under stress are generally more predictable. Knowing a subject's behavior in any one task is highly predictive of his behavior in the other two tasks. In addition, stress may affect the way we view the agency of our opponent. In our experiment, the behavior of stressed subjects was similar whether they were facing an objective probability or a strategic opponent. When facing an opponent, they expected the same behavior in games that are opposite in nature. One implication from this is that stress causes people to treat others as if they have less sophistication or less agency, which may have other ramifications in social settings.

The paper is organized as follows. Section 2 describes our experimental design and predictions, with particular emphasis on the methodological contributions. Section 3 analyzes the aggregate data in each task and treatment. Section 4 studies the effect of stress on decision-making both across and within tasks, which provides our main result pertaining to contextual blindness. Section 5 investigates how stress and task complexity affect reaction times. Section 6 concludes.

## 2. Design and procedures

### 2.1. Experimental design

We first describe our experimental design. Further details regarding implementation, timing, and exclusion criteria are relegated to Appendix A1.

#### 2.1.1. Stress inducement and hormonal analysis

To induce a stress response in our treatment group, we closely followed the protocol of the Socially Evaluated Cold Pressor Test (SECPT, Schwabe et al., 2008). This task requires subjects to place their hand in ice water while their face is video recorded. All 72 subjects in the stress group successfully passed our requirements for completing the SECPT. To measure hormonal changes, we followed the “passive drool” protocol provided by the laboratory that ran our assay analysis (ZRT Labs). Each subject was required to submit 3 saliva samples in order to collect data on their baseline, peak, and end cortisol levels. All samples were viable and were used to measure the amount of circulating cortisol.

#### 2.1.2. Timeline and saliva sample collection

Since stress responses widely vary across individuals, we followed most of the literature on stress (Preston et al., 2007; Lighthall et al., 2009; Van Den Bos et al., 2009) and implemented a between-subjects design, with *Control* and *Stress* subjects (such method also avoids learning and endowment effects). The timeline of the experiment was the following. First, we provided detailed instructions of the tasks and performed a comprehension quiz. Subjects submitted their “Baseline” saliva sample. Subjects in the control treatment started the tasks immediately after the Baseline sample, whereas subjects in the stress treatment performed the SECPT before starting the tasks. Twenty five minutes after the Baseline saliva sample, all subjects were instructed to stop making choices in the task, and we collected the “Peak” saliva sample. Subjects completed the remaining tasks along with a brief demographic survey. They were shown all their choices and outcomes and provided the “End” saliva sample. One outcome was then randomly chosen by the computer to be used for payment. The average intra- and inter-assay coefficients of variation were no greater than 7 and 8%, respectively.

**Figure 2:**
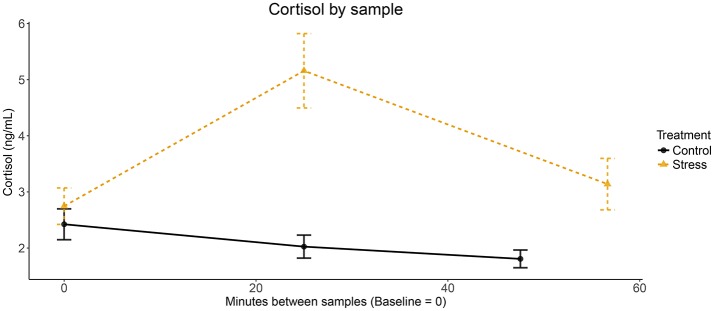
Cortisol levels over time.

The procedure had a limitation. Indeed, due to the absence of the SECPT task, the experiment took less time in the control treatment than in the stress treatment. This is reflected in Figure 2, where the average time between the Baseline and End saliva sample is 47.6 and 56.6 min, respectively. Ideally, the control treatment should have included a “placebo” task to replace the SECPT (e.g., hand immersion in warm water during 3 min) both to equalize the length and attention demand of the experiment and to have the saliva samples taken at approximately the same intervals.

#### 2.1.3. Participants and sessions

The study was reviewed by the University Park Institutional Review Board at the University of Southern California (UP-14-00663). Experiments were conducted at the Los Angeles Behavioral Economics Laboratory (LABEL) at the University of Southern California. To participate in the experiment, subjects could not eat, drink anything other than water, smoke, exercise, ingest caffeine, or chew gum within 1 h upon arriving at the laboratory. Subjects were also excluded if they had been asleep within 2 h prior to arriving at the lab or used any lip products at any time after 8 a.m. on the day of the experiment.

All sessions started at 3 p.m. and lasted no longer than 5:15 p.m. They had either 6 or 8 subjects with, at most, two more subjects of one gender in a session. We gathered data on a total of 144 subjects. One subject (stress group) was excluded due to a baseline cortisol 15 times the average of the sample, so our data is comprised of the choices of 143 subjects (71 stress, 66 female).

### 2.2. Tasks

Each subject made choices in three experimental tasks: individual lotteries **(LO)**, Stag Hunt games **(SH)**, and Hawk-Dove games **(HD)**. All three tasks have a *Safe* option *S* and a two-state *Risky* option, *R*_*H*_ and *R*_*L*_, so that *R*_*L*_ < *S* < *R*_*H*_. The inherent nature of risk in each task differs. **LO** is an individual choice problem, where the (objective) probability of earning *R*_*H*_, *p* ≡ Pr(*R*_*H*_), is known before the choice is made. **SH** and **HD** are two-person, simultaneous, non-cooperative games, where the probability of earning *R*_*H*_ depends on the choice of another subject in the room. In **SH**, the probability of earning *R*_*H*_ is *increasing* in the level of risk chosen by the other subject (a coordination game where risk-taking is a strategic complement), whereas in **SH** it is *decreasing* in the level of risk chosen by the other subject (an anti-coordination game where risk-taking is a strategic substitute). The basic structure of the tasks is summarized in Table 1,^4^.

**Table 1:**
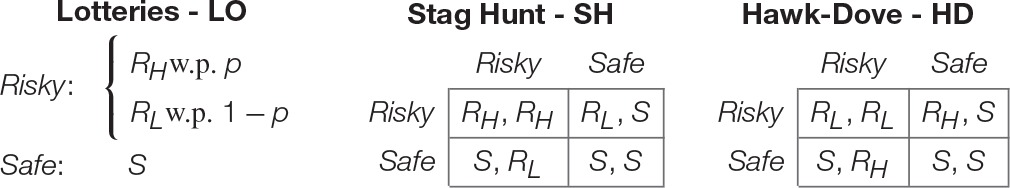
Experimental tasks.

To implement these three tasks, we construct the following novel design. In each round, subjects are given 100 tokens, that they must allocate between the *Safe* and *Risky* options (neutrally labeled “Option A” and “Option B” in the experiment). The computer then randomly selects a ball from an urn with 100 green and orange balls (see below). For any token allocation *x* (∈{0, …, 100}) to *Safe* and 100 − *x* to *Risky*, the payoff obtained by the subject is:

$$\frac{x}{100}S+\frac{100-x}{100}R_{H}\text{ if the computer draws a green ball}$$

$$\frac{x}{100}S+\frac{100-x}{100}R_{L}\text{ if the computer draws an orange ball}$$

In words, each token allocated to *Safe* yields $\frac{S}{100}$ whereas each token allocated to *Risky* yields either $\frac{R_{H}}{100}$ or $\frac{R_{L}}{100}$. As *x* decreases, the spread between the payoff if the computer draws a green and an orange ball increases. If the subject sets *x* = 100, she obtains *S* for sure. If the subjects sets *x* = 0, she obtains either *R*_*H*_ (green ball) or *R*_*L*_ (orange ball).

As described, for each token allocated to *Risky*, the probability of earning payoffs $\frac{R_{H}}{100}$ and $\frac{R_{L}}{100}$ are simply the proportion of green balls and orange balls in the computer's urn, respectively. The only difference between our three tasks **LO**, **SH**, and **HD** is the way in which the number of green and orange balls is determined:

- In **LO**, the number of green and orange balls is fixed and known (given by *p*).
- In **SH**, the number of green and orange balls is equal to the number of tokens that the participant with whom the subject is matched allocates to *Risky* and *Safe*, respectively.
- In **HD**, the number of green and orange balls is equal to the number of tokens that the participant with whom the subject is matched allocates to *Safe* and *Risky*, respectively.

In addition, in **SH** and **HD** subjects are told that their choice affects the number of green and orange balls in the urn of the participant with whom they are matched in the exact same way. That is, in **SH (HD)** the more tokens a subject allocates to *Risky*, the *more* (*less*) likely it is that the other participant earns *R*_*H*_.

Figure 1 provides screenshots of the **LO** (top), **HD** (bottom left) and **SH** (bottom right) tasks. At the top of the screen, the subject is told the current task (neutrally labeled as “Method 1,” “Method 2,” and “Method 3,” respectively). She is also reminded how the number of green and orange balls in her urn is determined. At the center of the screen, the subject can observe the parameters of the current round. In these three tasks, *S* = *$*21, *R*_*H*_ = *$*53 and *R*_*L*_ = *$*13. At the bottom of the screen, there is a slider that the subject can use to allocate her 100 tokens across *Safe* and *Risky*. As the subject moves the slider to test different token allocations, the earnings for each ball color are calculated and presented in real-time on the screen. In all three screenshots, the subject has set *x* = 29. After the subject is satisfied with the allocation of tokens, she has to click the “confirm” button to submit her choice.

Our experiment has two methodological contributions that we would like to emphasize. First, the contextual presentation of the three tasks is almost identical. Only the information concerning the determination of green and orange balls is changed. Capturing the inherently different natures of risk in such a symmetric way serves an important purpose: different behavior is likely to be only in response to the meaningful differences between these tasks, rather than to superficial differences in presentation or comprehension. Second, endowing subjects with 100 tokens that can be allocated across *Safe* and *Risky* can be used to measure “interior” behavior. In lotteries, it is analogous to portfolio diversification. In games, it is analogous to allowing subjects to play mixed strategies. In both cases, it provides more information than the standard binary choice method.

### 2.3. Payoff-variants, stakes, and equilibria

Subjects played a total of 48 rounds, 16 rounds of each task all with different payoffs. The experiment was broken up into blocks of 4 consecutive rounds of the same task, and all sessions started with a **LO** block, which was arguably simpler. Before each block, subjects were shown a screen reminding them that a new block was starting. This screen ensured that subjects would be aware of which task (**LO**, **SH**, or **HD**) they were playing next. For the games, subjects were randomly and anonymously rematched after each round. For the lotteries, they were playing an individual decision problem (the exact experimental instructions are in Appendix B). To avoid learning effects, subjects did not see the behavior of their partner nor the color of the ball drawn by the computer in each round. At the end of the 48 rounds, subjects observed all their choices and those of their partners. One round was randomly drawn by the computer and the outcome in that round was used for payment. Subjects earned an average of $31, with a minimum of $1 (twice) and a maximum of $53 (three subjects). In addition to these earning, all subjects were paid a $5 show-up fee.

We chose the payoffs in order to provide substantial variation in monetary stakes and equilibrium predictions. First, define:

(1) $$\Delta \equiv R_{H}-R_{L}$$

as a measure of the monetary *stakes*. For all tasks, we set Δ ∈ {10, 20, 30, 40}. In the analysis, we will refer to “low stakes” as Δ ∈ {10, 20} and “high stakes” as Δ ∈ {30, 40}. Second, given a triplet (*R*_*L*_, *S, R*_*H*_), the mixed-strategy Nash equilibrium of the **SH** game is:

(2) $$\alpha \equiv \frac{S-R_{L}}{R_{H}-R_{L}}$$

where α is the probability of choosing *Risky*. For each Δ, we choose (*R*_*L*_, *S, R*_*H*_) so that α ∈ {0.2, 0.4, 0.6, 0.8}. This gives 16 combinations of stakes and mixed equilibrium predictions in **SH**. Finally, notice that once we fix Δ, then α is proportional to *S* the payoff of the *Safe* option.

Notice that for a given triplet (*R*_*L*_, *S, R*_*H*_), the mixed-strategy Nash equilibrium of **HD** is:

(3) $$1-\alpha \equiv \frac{R_{H}-S}{R_{H}-R_{L}}$$

where 1−α is the probability of choosing *Risky*. Therefore, the same payoff-triplets as in **SH** provide also 16 combination of stakes (Δ ∈ {10, 20, 30, 40}) and mixed-strategy equilibria (1 − α ∈ {0.8, 0.6, 0.4, 0.2}) in **HD**. Last, we use the technique developed by Jessie and Kendall (2015) to select the payoffs in a way that the differences between games are only in the component that the Nash Equilibrium uses to make predictions. Table 2 provides a sample of eight games used in the experiment and Appendix A2 provides the entire list.

**Table 2:**
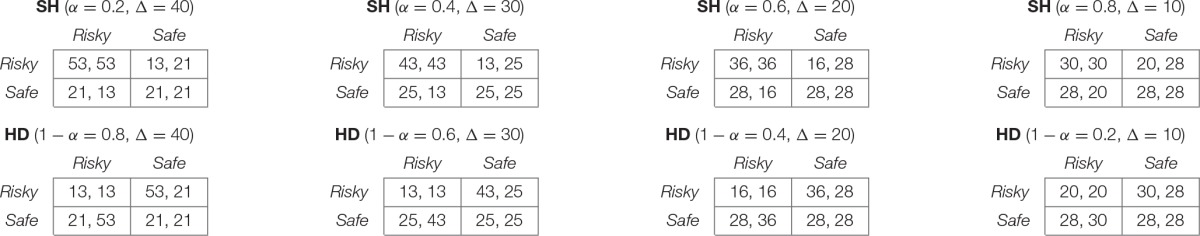
Examples of payoff-variants in **SH** and **HD** tasks.

Finally, to create the **LO** tasks, we choose the payoffs (*R*_*L*_, *S, R*_*H*_) of the **SH** and **HD** games corresponding to the extreme mixed-strategy Nash equilibria of the games: α = 0.2 and α = 0.8. Using these payoffs, we set the lottery probability of the high payoff *R*_*H*_ to *p* = 0.2 and *p* = 0.8. Creating four lotteries in this way for Δ ∈ {10, 20, 30, 40} yields a total of 16 **LO** tasks. Table 3 provides some examples of lotteries.

**Table 3:** Examples of payoff-variants in **LO** tasks.

**LO** (α = 0.2;	**LO** (α = 0.2;	**LO** (α = 0.8;	**LO** (α = 0.8;
Δ = 40; *p* = 0.8)	Δ = 30; *p* = 0.2)	Δ = 20; *p* = 0.8)	Δ = 10; *p* = 0.2)

*Safe*: 21 w.p. 1	*Safe*: 22 w.p. 1	*Safe*: 30 w.p. 1	*Safe*: 28 w.p. 1
*Risky*: 53 w.p. 0.8	*Risky*: 46 w.p. 0.2	*Risky*: 34 w.p. 0.8	Risky: 30 w.p. 0.2
13 w.p. 0.2	16 w.p. 0.8	14 w.p. 0.2	20 w.p. 0.8

### 2.4. Predictions

Our model has three parameters (Δ, α, *p*) in the **LO** tasks and two parameters (Δ, α) in the **SH** and **HD** tasks.

Predictions in **LO** are standard. Fixing the other two parameters, *Risky* becomes more attractive as *p* increases (first-order stochastic increase in the risky option) and α decreases (*S* closer to *R*_*L*_). The effect of Δ is less clear. For example, increasing Δ makes *Risky* more desirable when *p* = 0.8 and α = 0.2 and less desirable when *p* = 0.2 and α = 0.8.

Predictions in **SH** and **HD** are more subtle. By construction, in all 32 rounds there are two pure-strategy and one mixed-strategy equilibria. Subjects may move from one equilibrium to another, so behavior depends crucially on beliefs about the other player's action and comparative statics should be taken with a grain of salt. However, fixing the belief about the other player's constant, it seems intuitive that *Risky* is more attractive in both **SH** and **HD** as the sure payoff *S* becomes closer to *R*_*L*_, that is, as α decreases. Again, the effect of changes in the spread of payoffs Δ is more nuanced and depends on the position of *S*.

Finally, there are also interesting differences between **SH** and **HD**. SH is a coordination game, where risk-taking behavior is a strategic complement. This means that, holding constant the belief about the opponent, a decrease in α offers the subject more incentives to take risks. Furthermore, the subject realizes that the opponent also has more incentives to take risks, reinforcing the value of playing *Risky*. By contrast, **HD** is an anti-coordination game where risk-taking behavior is a strategic substitute. As α decreases, the subject has more incentives to choose *Risky* but realizes that the opponent has the same incentives, which decreases the value of risk-taking. Overall, strategic considerations make comparative statics significantly easier to evaluate when incentives of players are aligned **(SH)** than when they are not **(HD)**.

## 3. Aggregate results

### 3.1. Stress

Figure 2 shows the evolution of cortisol levels throughout the experimental sessions in both treatments. Each dot represents the average level of salivary cortisol samples (ng/mL) taken at baseline, peak, and end of the experiment. We report minutes on the x-axis. Note that the timing of the end sample was different across sessions and we represent the average number of minutes in each treatment. The control and stress groups start with statistically indifferent levels of average cortisol (2.42 vs. 2.75; two-sided Welch *t*-test, *p*-value = 0.133). The stress group experiences a large and statistically significant increase in average cortisol (2.75 vs. 5.16; *p*-value < 0.001). In comparison, the control group experiences a slight and statistically significant decrease in average cortisol (2.42 vs. 2.03; *p*-value = 0.022). Higher cortisol levels are also observed in the stress group in the end sample (1.81 vs. 3.14; *p*-value < 0.001).

### 3.2. Allocation between options

The average proportion of wealth invested in *Safe* is 0.63 in **LO**, 0.53 in **SH** and 0.65 in **HD**. Results between lotteries and games are not directly comparable. By contrast, results between the two games are comparable since the 16 rounds of **SH** involve the same payoff triplets (*R*_*L*_, *S, R*_*H*_) as the 16 rounds of **HD**. We notice a significantly lower allocation to *Safe* in **SH** than in **HD** < 0.001).

### 3.3. Testing the theory

#### 3.3.1. Behavior in lotteries

Choices in **LO** conformed to the theoretical predictions. Holding Δ constant, the proportion allocated to *Safe* increased as α increased and as *p* decreased for all stakes and in both treatments. Overall, subjects were (weakly) risk averse. They invested, on average, 97% of the endowment in *Safe* when the expected value of *Risky* was below the *Safe* option, against 70% when it was equal and 17% when it was above the *Safe* option^5^. Finally, the proportion in *Safe* was significantly lower in the low stakes rounds (Δ ∈ {10, 20}) compared to the high stakes rounds (Δ ∈ {30, 40}) under stress (*p*-value = 0.035) but only marginally in the control group (*p*-value = 0.051).

#### 3.3.2. Behavior in games

The proportion of wealth allocated to *Safe* varied with α as predicted in Section 2.4. In **SH** and keeping beliefs constant, increasing α makes *Safe* more attractive for a subject and, as the same logic applies for the partner, higher allocation rates in *Safe* are expected. Table 4 (left) shows that this is exactly how subjects behave for all stake levels. The average fraction allocated to *Safe* was significantly different between all pairs of α for all Δ (*p*-values < 0.05). In **HD** and keeping beliefs constant, increasing α (that is, decreasing 1−α) makes again *Safe* more attractive and should push more subjects to invest in *Safe*. However, they should expect their partner to also invest more in *Safe*, which should ultimately reduce the incentives to invest in that option. This implies that the response to an increase in α in **HD** should be less pronounced than in **SH**. Empirically, Table 4 (right) shows that increasing α made subjects invest significantly more in *Safe* for all pairs of α and all Δ (*p*-values < 0.05)^6^. Finally, we also computed for each individual the average increase in the fraction allocated to *Safe* between α = 0.2 and α = 0.8 in both **SH** and **HD**. We found a statistically higher increase in **SH** than in **HD** (0.56 vs. 0.43, *p*-value < 0.001), suggesting that subjects understood the difference between the strategic complementarity and the strategic substitutability of risk-taking in these two tasks. Last and as noted before, there is no particular reason to observe an aggregate effect of stakes in behavior. Empirically, we found none.

**Table 4:** Allocation to *Safe* as a function of α and Δ by game (pooled treatments).

**SH**	**Stakes** (Δ)				**HD**	**Stakes** (Δ)			
	40	30	20	10		40	30	20	10
α = 0.2	0.31	0.27	0.19	0.23	α = 0.2	0.49	0.49	0.36	0.41
α = 0.4	0.49	0.46	0.41	0.41	α = 0.4	0.59	0.58	0.55	0.52
α = 0.6	0.66	0.61	0.62	0.69	α = 0.6	0.73	0.72	0.72	0.78
α = 0.8	0.75	0.83	0.80	0.82	α = 0.8	0.83	0.83	0.89	0.89

Result 1. *On aggregate, subjects behave in accordance with our predictions: the allocation to the safe option is increasing in α in all three tasks and decreasing in p in lotteries. Changes in stakes have no systematic effect on behavior*.

## 4. Stress

### 4.1. Stress and tasks

**Figure 3:**
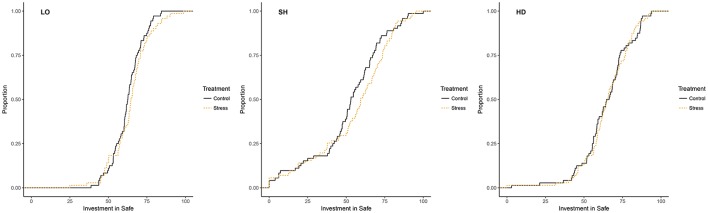
Distribution of average amounts in *Safe* by task and treatment.

We noted a slight increase in the average proportion allocated to *Safe* in the stress treatment in all tasks compared to the control treatment (0.64 vs. 0.63 in **LO**, 0.55 vs. 0.52 in **SH**, and 0.65 vs. 0.65 in **HD**). However, the differences were not statistically significant. As presented in Figure 3, the cumulative distribution functions of the average amounts allocated to *Safe* were also similar across treatments in all three tasks, with no statistically significant effect according the Kolmogorov-Smirnoff test (*p*-value = 0.31 in **LO**, *p*-value = 0.31 in **SH**, and *p*-value = 0.97 in **HD**). Overall, we found no evidence that stress affected behavior within each task.

The existing literature is ambiguous on this issue. Some studies have found that stress affects behavior in lotteries (Preston et al., 2007; Lighthall et al., 2009; Van Den Bos et al., 2009) whereas others found no effect of stress (von Dawans et al., 2012; Gathmann et al., 2014). Differences in responses to stress may be attributed to differences across studies in risk elicitation methods (BART, IGT, objective lotteries) and experimental procedures (presence/absence of incentives, hypothetical/real choices, different stressors). For instance, it may be that the emotional component contained in the BART experiment (anticipation of the balloon explosion and visual representation of such explosion) is responsible for shifts in behavior. Moreover, in BART and IGT subjects are typically *not* informed of the objective probabilities of the events. This ambiguity component may also trigger different thought processes that are differentially affected by stress (Buckert et al., 2014; Danese et al., 2017).

### 4.2. Stress and gender

In Table 5 we present the differences in allocation across gender. In the control condition, females allocate significantly more to *Safe* than males in **LO** and **SH** but not in **HD**. In the stress condition we find no significant gender differences in any task.

**Table 5:** Average allocation to *Safe* by gender, treatment and task.

	***Control***			***Stress***		
	**Female**	**Male**	**Difference [*p*-value]**	**Female**	**Male**	**Difference [*p*-value]**
**LO**	0.65	0.60	0.045	0.66	0.62	0.194
	(0.018)	(0.014)		(0.023)	(0.020)	
**SH**	0.59	0.46	0.015	0.60	0.51	0.143
	(0.039)	(0.037)		(0.042)	(0.040)	
**HD**	0.66	0.63	0.554	0.68	0.63	0.112
	(0.033)	(0.022)		(0.024)	(0.027)	

*Standard errors in parenthesis*.

Our data contribute to gender research in three ways. First, the fact that women take less risk in **LO** in the control group aligns with earlier literature (Charness and Gneezy, 2012). Second, finding males in the control group to be more cooperative in **SH** contributes to our understanding of gender differences in coordination games. However, we are hesitant to extrapolate about general inclinations to cooperate since, as suggested by Croson and Gneezy (2009), gender differences seem to be highly sensitive to context. Finally, since the only significant gender differences are found in the control group, we conclude that stress has the capability to diminish differences between genders.

### 4.3. The effect of stress on the relationship between tasks

Our next question is whether the willingness of individuals to choose *Risky* is correlated across tasks. On the one hand, it seems natural that subjects who are less risk-averse, that is, those who invest more in *Risky* in **LO** (individual lotteries with objective probabilities) are also expected to take more risks in games. On the other hand, this may not be necessarily true since our games have multiple equilibria, so risk-taking in **SH** and **HD** depends crucially on beliefs about the other player's behavior. Furthermore, the two games are fundamentally opposite in the optimal reaction to the other player's choice (coordination vs. anti-coordination). Table 6 presents the Pearson correlation coefficient (ρ) of the proportion allocated to *Safe* by individuals across tasks, both in the control (left panel) and stress (right panel) conditions.

**Table 6:** Correlation of individual risk taking behavior across tasks by treatment.

	***Control***		***Stress***	
	**LO**	**SH**	**LO**	**SH**
**SH**	0.347^**^	–	0.416^***^	–
**HD**	0.147	0.117	0.461^***^	0.497^***^

* p < 0.05;

** p < 0.01; and

*** *p < 0.001*.

In the control condition, the amount allocated to *Safe* in **LO** is significantly correlated with the amount allocated to *Safe* in **SH**, suggesting that risk attitude is a reasonably good predictor of behavior in the coordination game. This finding aligns with previous studies showing a correlation between **LO** and **SH** choices (Heinemann et al., 2009; Chierchia and Coricelli, 2015). By contrast, the control condition shows no significant correlation between **LO** and **HD** or between **SH** and **HD**. This may not be surprising given the previous research showing that these tasks activate different areas of the brain (Ekins et al., 2013; Nagel et al., 2014).

By contrast, in the stress condition, the amounts allocated to *Safe* are significantly correlated across all tasks. Correlations are also stronger, suggesting that risk-taking under stress is very similar across tasks, irrespective of the situation. This important result indicates that, even though stress did not have an effect on the overall distribution of risk taking in the population across tasks, it did affect intra-personal decisions. The result was confirmed by a set of robust regressions reported in Table 7, which suggests a stronger relationship between the amount allocated to *Safe* in **LO**, **SH** and **HD** under stress than in the control treatment. This effect will be corroborated with the trial-by-trial regression analysis.

**Table 7:** Robust regression of the average investment in *Safe* in **SH** and **HD** on the average investment in the safe option in lotteries (*Safe*-***LO***) by treatment.

	***Control***		***Stress***	
	**SH**	**HD**	**SH**	**HD**
*Safe-**LO***	0.94^**^	0.52^*^	1.06^***^	0.78^***^
*Constant*	−4.41	32.86^*^	−9.33	16.84^*^
Robust SE	17.7	11.92	16.94	10.13
Adj. *R*^2^	0.168	0.126	0.323	0.460

* p < 0.05;

** p < 0.01; and

*** *p < 0.001*.

We then compared the correlation coefficients across conditions by assessing statistical significance of the Fisher's r to z transformations. We found that the correlation between **LO** and **SH** are not significantly different between control and stress conditions. By contrast, correlations between **LO** and **HD** and between **SH** and **HD** respectively are significantly different (with respective *p*-values of 0.040 and 0.012). This result further supports the finding that subjects under stress make choices that are more similar across tasks than subjects in the control treatment.

A possible explanation for this result is that subjects under stress (and only those subjects) exhibit contextual blindness, that is, they ignore the context that distinguishes these three tasks. Indeed, **LO** measures an individual's propensity to take risks which has no social context. **SH** captures a tension between risk and cooperation whereas **HD** captures a tension between risk and aggression. The experiment was designed so that these contexts were the only difference between tasks. Table 6 reveals that the behavior of stressed subject when faced with an objective probability over earnings was strongly and positively correlated with their behavior when faced with a strategic opponent, even if games were opposite in nature. For control subjects there was only a relationship between **LO** and **SH**. In other words, control subjects responded more to the differing contexts than stressed subjects. One implication is that the choices of subjects under stress are generally more predictable: knowing the average amount a subject invests into *Safe* in any one task provides significant information about behavior in the other two.

We also ran OLS regressions of the trial-by-trial amounts allocated to *Safe* for each game and in each condition. We used as regressors the individual average amount allocated to *Safe* in **LO** (which captures the risk attitude of each individual), and dummies for stakes (1 = High stakes), for the position of *S* relative to *R*_*L*_ and *R*_*H*_ (α), and for gender (1 = Male). We constructed a fixed effect model by including a dummy variable for each individual. The results are compiled in Table 8.

**Table 8:** OLS of investment in *Safe* in **SH** and **HD** including fixed effects.

	***Control***		***Stress***	
	**SH**	**HD**	**SH**	**HD**
Lottery	0.70^*^	0.23	0.78^***^	0.55^***^
	(0.27)	(0.20)	(0.22)	(0.13)
*Male*	−10.07	−1.27	−5.52	−3.64
	(5.29)	(3.97)	(5.46)	(3.35)
*High Stakes*	0.28	0.05	4.87^**^	3.42^*^
	(1.68)	(1.54)	(1.60)	(1.46)
α = 0.8	55.3^***^	39.3^***^	54.9^***^	44.6^***^
	(2.37)	(2.18)	(2.27)	(2.07)
α = 0.6	40.4^***^	30.6^***^	38.9^***^	29.6^***^
	(2.37)	(2.18)	(2.27)	(2.07)
α = 0.4	17.5^***^	13.1^***^	20.9^***^	11.3^***^
	(2.37)	(2.18)	(2.27)	(2.07)
Constant	−15.4	30.23^*^	−23.2	8.9
	(18.0)	(13.5)	(15.1)	(9.3)
Observations	1,152	1,152	1,136	1,136
FE groups	72	72	71	71
df	9	9	9	9
Log-likelihood	−5,559	−5,447	−5,429	−5,300
BIC	11,182	10,957	10,922	10,663

*Standard errors in parenthesis*.

* p < 0.05;

** p < 0.01; and

*** *p < 0.001*.

In the *Control* condition, the average allocation to *Safe* in **SH** is predicted by the behavior in **LO**, but the average allocation in **HD** is not. In the *Stress* condition, the average amounts allocated to *Safe* in both **SH** and **HD** are highly predicted by behavior in **LO**. These regressions further confirm the contextual blindness result^7^. We also notice that gender has no explanatory power and that the allocation to the safe choice is increased for high stakes, but only in the *Stress* condition.

To better assess the significance of the effect of stress in **HD**, we ran a regression of the trial-by-trial amounts allocated to *Safe* in **HD** in both conditions on the same regressors as before as well as the individual difference in cortisol between baseline and peak (Δ*Cortisol*) and an interaction term between that measure and the average allocation to *Safe* in **LO**^8^. For comparison, we ran the same regression for **SH** as well. This exercise tests directly whether the coefficients of the average allocation to *Safe* in **LO** in the previous table are significantly different across treatments. The results are reported in the first two columns of Table 9. The absence of a significant interaction in the case of **SH** confirms that the amount allocated to *Safe* in **LO** does not predict differentially behavior in **SH** across conditions. By contrast, the interaction term is significant in the case of **HD**, the contribution of the amount allocated to *Safe* in **LO** to behavior in HD differs across conditions. We finally ran a full regression over both games using a dummy variable for our games (1 = **SH**). The results are reported in the last column of Table 9. The fact that the three way interaction between the average allocation to *Safe* in **LO**, the treatment and the increase in cortisol is significant indicates that the interaction between *Safe* in **LO** and Stress is significantly different across games. The regression also shows a subtle interaction between cortisol increase and games: subjects who exhibit a higher increase in cortisol level tend to increase more their investment to *Safe* in **HD**.

**Table 9:** OLS with interactions of investment in *Safe* in **SH** and **HD** including fixed effects.

	**SH**	**HD**	**All games**
*Lottery*	0.73^***^	0.28^*^	0.25
	(0.19)	(0.12)	(0.13)
*ΔCortisol*	−0.17	−7.81^**^	−8.33^*^
	(4.81)	(3.22)	(3.33)
*Game (**SH** = 1)*	–	–	−44.16^***^
			(5.36)
Lottery^*^ΔCortisol	0.01	0.12^**^	0.13^**^
	(0.07)	(0.05)	(0.05)
Lottery^*^Game	–	–	0.51^***^
			(0.08)
ΔCortisol^*^Game	–	–	8.67^***^
			(2.19)
Lottery^*^ΔCortisol^*^Game	–	–	−0.13^***^
			(0.03)
*Male*	−7.93^*^	−3.64	−5.79^*^
	(3.85)	(2.58)	(2.52)
*High Stakes*	2.56^*^	1.72	2.14^*^
	(1.16)	(1.06)	(0.86)
α = 0.8	55.11^***^	41.90^***^	48.50^***^
	(1.64)	(1.50)	(1.21)
α = 0.6	39.66^***^	30.12^***^	34.89^***^
	(1.64)	(1.50)	(1.21)
α = 0.4	19.19^***^	12.17^***^	15.68^***^
	(1.64)	(1.50)	(1.21)
*Constant*	−19.44	26.94^**^	25.83^**^
	(12.68)	(9.3)	(8.73)
Observations	2,288	2,288	4,576
FE groups	143	143	143
df	11	11	15
Log-likelihood	−11,007	−10,764	−22,037
BIC	22,099	21,613	44,200

*Standard errors in parenthesis*.

* p < 0.05;

** p < 0.01; and

*** *p < 0.001*.

### 4.4. Cluster analysis

The fact that stress does not have any visible effect on aggregate behavior (Section 4.1) but reduces gender differences (Section 4.2) and impacts the relationship between tasks (Section 4.3) is puzzling. We therefore decided to study in more detail the behavior of individuals across the three tasks.

**Figure 4:**
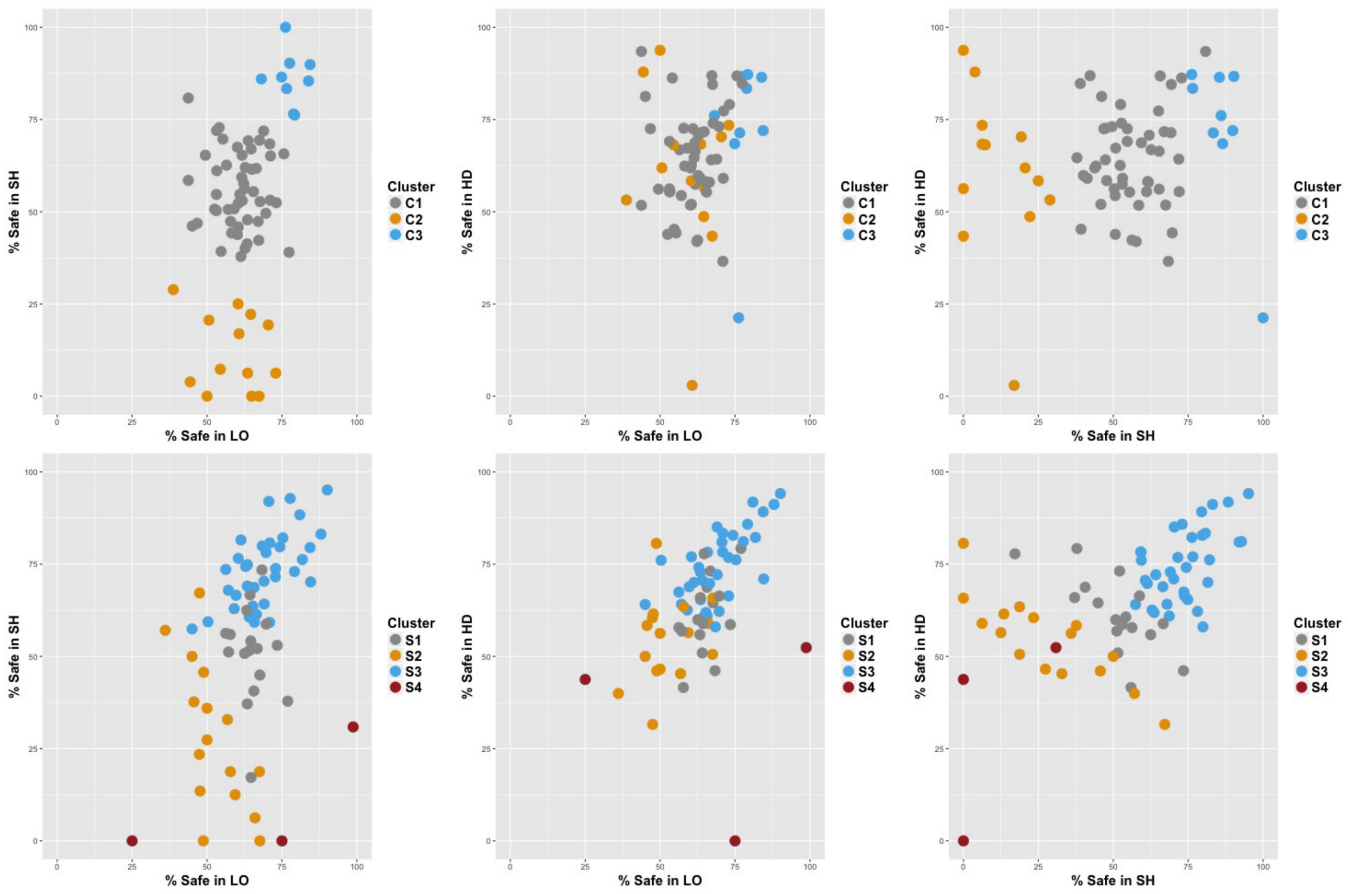
Representation of choices by cluster in the Control **(Top)** and Stress **(Bottom)** conditions.

We conducted a cluster analysis in each condition to group subjects according to their average allocation to *Safe* in each task. We retained a model-based clustering method to identify the clusters present in our population. A wide array of heuristic clustering methods are commonly used but they typically require the number of clusters and the clustering criterion to be set ex-ante rather than endogenously optimized. Mixture models, on the other hand, treat each cluster as a component probability distribution. Thus, the choice between numbers of clusters and models can be made using Bayesian statistical methods (Fraley and Raftery, 2002). We implemented our model-based clustering analysis with the Mclust package in R (Fraley and Raftery, 2006). We considered ten different models with a maximum of nine clusters each, and retained the cluster combination that yielded the minimum Bayesian Information Criterion (BIC). In the *Control* condition, the best model consisted of three clusters (C1, C2, and C3). In the *Stress* condition, four different clusters best summarized behavior (S1, S2, S3, and S4). Table 10 summarizes the descriptive statistics in each cluster. Figure 4 provides a visual representation of the clusters across treatments^9^.

**Table 10:** Endogenous clusters in each condition (standard errors in parenthesis).

	***Control***				***Stress***			
	**C1**	**C2**	**C3**		**S1**	**S2**	**S3**	**S4**
Male/Female	29/21	9/4	0/9		12/5	10/6	15/20	2/1
% *Safe* in **LO**	60.9	58.7	77.7		65.1	52.6	69.1	66.3
	(1.11)	(2.87)	(1.62)		(1.30)	(2.25)	(1.74)	(21.7)
% *Safe* in **SH**	56.0	12.1	86.0		50.9	27.9	73.4	10.3
	(1.49)	(2.88)	(2.42)		(3.13)	(5.02)	(1.71)	(10.3)
% *Safe* in **HD**	64.2	60.5	72.5		61.4	54.5	74.7	32.0
	(1.87)	(6.23)	(6.86)		(2.46)	(2.89)	(1.64)	(16.2)

In the *Control* condition, the majority of the subjects (C1) exhibited the typical behavior: they invested similar proportions in the *Safe* asset in **LO** and **HD** and less in **SH**, suggesting large homogeneity across subjects in this treatment. A few individuals (C2) were an extreme version of this typical play, with overly risky behavior in **SH**. Finally, a minority of all female subjects (C3) allocated significantly more to *Safe* in **LO** and **HD**, but especially in **SH**. This group was responsible for the gender effect detected in **LO** and **SH** in the control condition.

In the *Stress* condition, there were three main clusters (S4 consists of 3 outliers), similar to the clusters obtained in the control condition. Cluster S1 was the analog of C1, while S2 was similar to C2, except for a safer proportion of choices in **SH**. However, half of the subjects were now grouped in S3, a cluster similar to C3. These subjects allocated a large fraction of their endowment to *Safe* in all tasks. S3 had also the particularity that allocations were extremely similar across tasks (69.1–74.7% with low standard errors). These subjects were responsible for strengthening the relationship between tasks. Moreover, there was no gender supremacy in that cluster, causing the gender effect observed in the control condition to disappear under stress.

Result 2. *Aggregate behavior is similar across treatments whereas individual choices are affected by stress. A significant fraction of participants in the stress condition are subject to contextual blindness, choosing a similar allocation independently of the task*.

## 5. Reaction times

### 5.1. Task difficulty

In Table 11 we report the average reaction time (RT) in seconds separated by task and treatment.

**Table 11:** Reaction time by task and treatment.

	**LO**	**SH**	**HD**	**All**
***Control***	25.6	24.2	28.2	26.0
	(0.71)	(0.61)	(0.62)	(0.37)
*Stress*	27.3	25.7	31.1	28.0
	(0.71)	(0.65)	(0.73)	(0.40)
Difference	0.087	0.097	0.002	<0.001
[*p*-value]				

*Standard errors in parenthesis*.

Making choices took more time under stress across all tasks, although the effect was mostly due to **HD**. We also found that RT were longer in **HD** compared to **SH** irrespective of the treatment (*p* < 0.001), consistent with the idea that the anti-coordination game is more complex to evaluate than the coordination game.

### 5.2. Attention in lotteries

As reflected in Table 12, risky options with expected value below the safe alternative (EV < *S*) were quickly discarded. Subjects took significantly more time to choose when the expected value of the risky option was equal (EV = *S*) or greater (EV>*S*) than the safe option (*t*-test, *p*-value < 0.01 for all paired comparisons in *Control* and *Stress* treatments). For the more complex lotteries (EV>*S*), subjects took slightly more time under stress, although not significantly so.

**Table 12:** Reaction time in lotteries by treatment and expected value of lottery (EV).

	***Control***	***Stress***	**Difference [*p*-value]**
EV < *S*	19.8	19.6	0.937
	(1.51)	(1.59)	
EV = *S*	28.6	30.3	0.420
	(1.59)	(1.51)	
EV > *S*	25.6	28.7	0.173
	(1.51)	(1.70)	

### 5.3. Attention in games

Table 13 presents the reaction times in **SH** and **HD** as a function of the parameters of the games, α and Δ.

**Table 13:** Reaction time in games as a function of α and Δ.

	α				Δ	
	0.2	0.4	0.6	0.8	**High**	**Low**
**SH**						
*Control*	24.1	28.2	22.7	21.6	27.7	20.6
	(1.74)	(1.76)	(1.53)	(1.49)	(1.64)	(1.21)
*Stress*	26.6	30.5	23.8	21.6	28.5	22.9
	(1.72)	(1.93)	(1.62)	(1.68)	(1.64)	(1.47)
**HD**						
*Control*	29.7	33.5	28.4	21.8	31.4	25.0
	(1.54)	(1.86)	(1.75)	(1.31)	(1.54)	(1.23)
*Stress*	35.1	34.8	31.6	24.2	35.0	27.2
	(1.94)	(1.93)	(2.24)	(1.74)	(1.82)	(1.36)

In **SH**, we found that RT were shorter for higher α: shortest at α = 0.8 and longest at α = 0.4 in both conditions (*t*-tests of difference, *p* < 0.01 in both conditions). We also found that RT were longer in high stakes than in low stakes rounds (*t*-test of difference, *p* < 0.001 in *Control* and *p* = 0.012 in *Stress*). The trend was identical in **HD**, with shortest RT at α = 0.8 and longest at α = 0.4 in the control group and α = 0.2 in the stress group (*t*-tests of difference, *p* < 0.001 in both conditions). RT were also longest in high stakes trials (*t*-test of difference, *p* < 0.001 in both groups). It is unclear why α significantly affects reaction times in the games. In both **SH** and **HD**, increasing α makes the safe option relatively more valuable. It is plausible that *Safe* becomes easier to evaluate as it becomes more attractive, resulting in a quicker response. As for stakes, we conjecture that subjects find the decision to be more important (hence, more worthy of attention) when, other things being equal, the set of payoffs is more spread out. In any case, the consistency of the reaction time comparative statics across games and conditions is remarkable and deserves further investigation. Finally, in **SH** there was no effect of stress. In **HD**, there was an increase in RT under stress only when α = 0.2 (*p* = 0.030) and when stakes were high (*p* = 0.015), suggesting an interaction between game complexity and difficulty to evaluate alternatives. It is also consistent with studies showing that stress affects working memory and executive decision-making. High levels of cortisol have been associated with more errors in card sorting tasks meant to measure executive functioning (McCormick et al., 2007) as well as O-span and backwards digit-span tasks meant to measure working memory (Schoofs et al., 2009). While our finding reflects the intuition behind results showing stressed subjects performing worse on more complicated tasks (Schoofs et al., 2009), our contribution shows that more complicated decisions also take longer (in our setting, there are no right or wrong decisions). This finding illustrates an important difference between how stressed subjects reach decisions in strategic games vs. in working memory or executive functioning tasks.

We then conducted a mixed effect OLS regression to better analyze the contribution of each effect to reaction times in both games. For both **SH** and **HD**, we regressed reaction times on a Treatment dummy (1 = *Stress*), a Gender dummy (1 = Male), a Stakes dummy (1 = High stakes), and dummies identifying the level of α in each round. The results are reported in Table 14. They confirm the effect of high stakes and α levels reported above. Stress and gender did not have significant effects.

**Table 14:** OLS of decision time in **SH** and **HD** including fixed effects.

	**SH**	**HD**
*Stress*	1.64	2.94
	(2.00)	(2.00)
*Male*	−2.24	0.141
	(2.00)	(2.00)
*High Stakes*	6.38^***^	7.02^***^
	(0.74)	(0.80)
α = 0.8	−3.69^***^	−9.08^***^
	(1.04)	(1.13)
α = 0.6	−2.02	−2.49^*^
	(1.05)	(1.14)
α = 0.4	4.04^***^	2.18
	(1.04)	(1.14)
Constant	22.52^***^	27.02^***^
	(1.91)	(1.93)
Observations	2,260	2,227
FE groups	143	143
df	9	9
Log-likelihood	−9,810	−9,831
BIC	19,689	19,731

*Standard errors in parenthesis*.

* p < 0.05;

** p < 0.01; and

*** *p ≤ 0.001*.

Result 3. *Reaction times are higher in the conceptually more difficult game **HD**, in the more complex rounds of **LO**, when stakes are high and when the safe option is intrinsically less attractive in **SH** and **HD**. Stress (weakly) increases reaction times in those cases*.

## 6. Discussion

In this paper, we examined the effect of stress on decision-making in three tasks: lotteries, Stag Hunt games, and Hawk-Dove games. Previous experiments and neuro-imaging studies suggest that people are responsive to differences in incentives across these tasks, which aligns with our control group. However, a significant portion of subjects under stress do not respond to these different incentives, which we interpret as contextual blindness.

The results contribute to our understanding of the complex relationship between stress and decision-making. In this regard, we found both conflicting and confirming evidence. Unlike some of the recent literature on lottery choice, in our study we did not find that stress had a systematic effect on any of the three tasks. However, our main finding of contextual blindness fits in well with previous work on stress inducing habituation with regard to cognitive inflexibility.

Stress-induced contextual blindness is demonstrated by a predictable pattern where subjects who choose to be relatively risk-seeking in one context also choose to be relatively risk-seeking in other, radically different ones. This predictability can be leveraged in order to reach desirable outcomes in coordination games either through directly modulating stress or by optimizing the pairing of players and games. For example, placing under stress two subjects who are risk-takers in lotteries may encourage them to be risk-seeking in Stag Hunt, therefore promoting the payoff-dominant equilibrium outcome. Alternatively, in settings where subjects need to be paired together to play coordination games, risk-preference can serve as a guide to create optimal subject-pairings in stressful circumstances. In Stag Hunt situations, optimal pairings would combine subjects with similar risk-seeking behavior in lotteries whereas in Hawk-Dove situations, optimal pairings would combine subjects with opposite risk preferences. Practical applications include team formation in military operations with limited communication.

Finally, it is surprising to observe similar attitudes when facing another individual and a lottery draw. The extent to which contextual blindness contributes to an attributed loss of opponents' agency is unclear. Subjects under stress have been shown to treat other players as less strategic decision-makers (Leder et al., 2013), but this is different from treating them as probabilistic outcomes. Further research may disentangle how stress modulates the level of autonomy attributed to other players. It may be that stress makes humans less likely to incorporate the *intention* of an action, which would have important implications in social contexts.

## Author contributions

IB, JC, and RK contributed to all aspects of this project equally.

### Conflict of interest statement

The authors declare that the research was conducted in the absence of any commercial or financial relationships that could be construed as a potential conflict of interest.

## Footnotes

^1^For example, subjects under stress are less accurate at identifying visual cues located on the periphery of their vision, but these same subjects are actually *more accurate* than their non-stressed counterparts at identifying cues directly in front of them (Hockey, 1970).

^2^A differential effect across gender is not surprising since, in general, stress is theorized to affect men and women differently (Taylor et al., 2000).

^3^There is also a literature relating stress to behavior in multi-person games. However, it is only tangentially related to our work as it focuses mainly on the effect of stress on prosocial or anti-social behavior (see Buchanan and Preston, 2014; Van Den Bos and Flik, 2015 for summaries).

^4^As it is well-know, SH is a coordination game with two pure-strategy equilibria (*Safe*-*Safe* and *Risky*-*Risky*) and one mixed-strategy equilibrium whereas **HD** is an anti-coordination game with two pure-strategy equilibria (*Safe*-*Risky* and *Risky*-*Safe*) and one mixed-strategy equilibrium.

^5^Since virtually no subject exhibited risk-loving preferences, the four **LO** rounds where *Risky* has lower expected value than the *Safe* option contain no extra information. As a robustness check, we conducted the entire analysis of the paper without these four rounds. All the results were statistically identical.

^6^Recall that in **SH**, α is the probability of playing *Risky* in the mixed strategy equilibrium. In **HD**, 1−α is the probability of playing *Risky* in the mixed strategy equilibrium.

^7^We also ran the same OLS regressions with the behavior in the other game as an extra regressor. Results and significance were very similar. Furthermore, and confirming the results in Table 6, the new variable had a positive and significant coefficient in the *Stress* regressions and a positive but not significant coefficient in the *Control* regressions. Notice that a two-censored non-linear Tobit model would allow for censoring at 0 and 100 but requires analysis at the subject-average level since it cannot account for subject-level fixed effects. The average data was rarely censored at either 0 or 100 which makes such a model inappropriate.

^8^The previous analysis only makes a qualitative comparison of the association between the allocation to Safe in **HD** and **SH** and the average allocation to Safe in **LO** in the two conditions. A formal analysis of the interactions between money allocation, games and conditions within the same model allows to directly compare the strength of the across conditions and games (see Nieuwenhuis et al., 2011).

^9^To better represent the information, we do not use three-dimensional graphs. Instead, we provide projections of each pair of tasks separately.
