# Supplementary material for: Stress Induces Contextual Blindness in Lotteries and Coordination Games
Source: Front Behav Neurosci. 2017 Dec 11;11:236. doi: 10.3389/fnbeh.2017.00236 (PMC5732178; doi:10.3389/fnbeh.2017.00236)
Supplement: Supplementary file 1 [file DataSheet1.pdf]

Appendix A1: Details of experimental design

SECPT utilization and exclusion criteria

For this task, each subject had an elastic band placed on their left arm slightly above the protrusion where the ulna connects to the wrist. Subjects were required to spread their fingers and place their left hand in ice water at 4 to 6 degrees Celsius to a depth that reached the elastic band. In addition, subjects were required to look directly into a camera mounted on top of their computer screen which displayed their face while their hand was in the water. Subjects were told that their facial expressions would be recorded, which we did. One experimenter was assigned to watch a set of two experimental subjects to ensure they kept their fingers spread open, their hand at a depth reaching the elastic band, and their face looking directly into the camera. Subjects were told that there was a 'required minimum amount of time' that they needed to follow these instructions 'in order to continue with the experiment.' Otherwise, they would be paid the show up fee ($5) and excluded from the experiment. Each subject was told that they only had 'one opportunity to keep your hand in the water for a long enough time to pass the minimum threshold.' The minimum threshold was 30 seconds, which was not known to the subjects. Instead, subjects were told that if they kept their hand in the water for 3 minutes they would be 'guaranteed to pass this portion of the experiment and will be allowed to continue.' All 144 subjects who were subjected to this SECPT passed. 140 of which kept their hands in the water for the full 3 minutes (while the other 4 passed the 30 second threshold).

Saliva collection protocol

Each saliva sample was collected as passive drool samples where subjects spit directly into a test tube to a level of at least 2.5 mL per sample. Immediately after collection, samples were stored in a freezer below -20 degrees Celsius. Within seven days of collection, samples were shipped in frozen shipping containers to ZRT laboratories for testing.

Further experimental details

Participants were required to stay in the room for the entirety of the experiment. Two subjects (from different sessions) needed to leave during the experiment. In these sessions another subject was randomly chosen to be excluded in order to maintain an even number of subjects. Each session was gender-mixed so that the worst gender imbalance would be two more of one gender than the other. This was done to avoid concerns that people behave differently when they know what gender they are playing against. Participants were randomly determined to be a control or treatment subjects before the start of a

session. Before entering the lab, subjects were instructed to sign an informed consent and to drink an 8 ounce bottle of water (to ensure clean saliva tests). Subjects were paid in private as they left the laboratory. Our data consists of 20 sessions.

Pre-experiment survey (to ensure conformity with recruiting instructions)

Please take this short survey before we begin the experiment:

At what time did you wake up most recently today? ________________

Have you eaten any food today? Yes / No
If Yes, when was the last time you ate anything? ________________

Have you drank anything other than water today? Yes / No
If Yes, when was the last time drank anything?________________

Have you exercised rigorously today? Yes / No
If Yes, when was the last time you exercised?________________

Have you smoked anything today? Yes / No
If Yes, when was the last time that you smoked?________________

Have you chewed gum today? Yes / No
If Yes, when was the last time that you chewed gum?________________

Have you ingested caffeine today? Yes / No
If Yes, when was the last time that you ingested caffeine?________________

Have you used any products on your lips today (such as chapstick, lip balm, lip stick, lip gloss, or sunscreen)? Yes / No
If Yes, when was the last time that you used any of these products?________________

I acknowledge that the answers that I have provided are accurate according to my memory.

Signature________________

Appendix A2: List of payoffs in all 48 rounds

Lottery tasks - **LO** (16 rounds)

SH40.2	Lottery	
	21	53, 13
	80 G; 20 O	

SH40.2	Lottery	
	21	53, 13
	20 G; 80 O	

SH40.8	Lottery	
	33	41, 1
	80 G; 20 O	

SH40.8	Lottery	
	33	41, 1
	20 G; 80 O	

SH30.2	Lottery	
	22	46, 16
	80 G; 20 O	

SH30.2	Lottery	
	22	46, 16
	20 G; 80 O	

SH30.8	Lottery	
	31	37, 7
	80 G; 20 O	

SH30.8	Lottery	
	31	37, 7
	20 G; 80 O	

SH20.2	Lottery	
	24	40, 20
	80 G; 20 O	

SH20.2	Lottery	
	24	40, 20
	20 G; 80 O	

SH20.8	Lottery	
	30	34, 14
	80 G; 20 O	

SH20.8	Lottery	
	30	34, 14
	20 G; 80 O	

SH10.2	Lottery	
	25	33, 23
	80 G; 20 O	

SH10.2	Lottery	
	25	33, 23
	20 G; 80 O	

SH10.8	Lottery	
	28	30, 20
	80 G; 20 O	

SH10.8	Lottery	
	28	30, 20
	20 G; 80 O	

Stag Hunt tasks - **SH** (16 rounds)

SH40.2	B	A
B	53, 53	13, 21
A	21, 13	21, 21

SH40.4	B	A
B	49, 49	9, 25
A	25, 9	25, 25

SH40.6	B	A
B	45, 45	5, 29
A	29, 5	29, 29

SH40.8	B	A
B	41, 41	1, 33
A	33, 1	33, 33

SH30.2	B	A
B	46, 46	16, 22
A	22, 16	22, 22

SH30.4	B	A
B	43, 43	13, 25
A	25, 13	25, 25

SH30.6	B	A
B	40, 40	10, 28
A	28, 10	28, 28

SH30.8	B	A
B	37, 37	7, 31
A	31, 7	31, 31

SH20.2	B	A
B	40, 40	20, 24
A	24, 20	24, 24

SH20.4	B	A
B	38, 38	18, 26
A	26, 18	26, 26

SH20.6	B	A
B	36, 36	16, 28
A	28, 16	28, 28

SH20.8	B	A
B	34, 34	14, 30
A	30, 14	30, 30

SH10.2	B	A
B	33, 33	23, 25
A	25, 23	25, 25

SH10.4	B	A
B	32, 32	22, 26
A	26, 22	26, 26

SH10.6	B	A
B	31, 31	21, 27
A	27, 21	27, 27

SH10.8	B	A
B	30, 30	20, 28
A	28, 20	28, 28

Hawk-Dove tasks - **HD** (16 rounds)

HD40.8	B	A
B	13, 13	53, 21
A	21, 53	21, 21

HD40.6	B	A
B	9, 9	49, 25
A	25, 49	25, 25

HD40.4	B	A
B	5, 5	45, 29
A	29, 45	29, 29

HD40.2	B	A
B	1, 1	41, 33
A	33, 41	33, 33

HD30.8	B	A
B	16, 16	46, 22
A	22, 46	22, 22

HD30.6	B	A
B	13, 13	43, 25
A	25, 43	25, 25

HD30.4	B	A
B	10, 10	40, 28
A	28, 40	28, 28

HD30.2	B	A
B	7, 7	37, 31
A	31, 37	31, 31

HD20.8	B	A
B	20, 20	40, 24
A	24, 40	24, 24

HD20.6	B	A
B	18, 18	38, 26
A	26, 38	26, 26

HD20.4	B	A
B	16, 16	36, 28
A	28, 36	28, 28

HD20.2	B	A
B	14, 14	34, 30
A	30, 34	30, 30

HD10.8	B	A
B	23, 23	33, 25
A	25, 33	25, 25

HD10.6	B	A
B	22, 22	32, 26
A	26, 32	26, 26

HD10.4	B	A
B	21, 21	31, 27
A	27, 31	27, 27

HD10.2	B	A
B	20, 20	30, 28
A	28, 30	28, 28

Appendix B: Experimental instructions

Welcome to this experiment at USC. Thank you for signing up.

You are about to participate in a study of decision-making, and you will be paid for your participation in cash. The amount you earn for participating in this experiment depends partly on your decisions, partly on the decisions by other subjects in the room, and partly on chance. There are several parts to this experiment, and your earnings will be totaled up and paid to you privately in the other room at the end of the experiment. This experiment is scheduled to last for 2 hours.

As a reminder, your participation in this experiment is voluntary, and you may leave the room at any time. If you choose to leave the room before the experiment is over, you will only be paid the $5 show-up fee and nothing that you have earned during the experiment.

Please turn off your cell phone. It is important that you do not communicate with any other participants in the room during the experiment.

If you have a question about what you are being instructed to do in this experiment, please raise your hand and an experimenter will come over to your station. We encourage questions that help clarify how the experiment works. However, we cannot answer questions about how you should make choices in the experiment.

As a reminder, this experiment does not contain any deception (lying). The way we describe the experiment and your earnings is the exact way that you will be paid.

First, we will go through some instructions detailing what is expected of you during the experiment. This will include some examples and practice questions. You will not be paid according to these practice questions and the exact practice questions will never be asked as part of the decision periods of the experiment. After the instructions, you will participate in the decision periods where you will be paid according to your choices. You will be reminded when the instructions and practice have concluded and the decision periods are about to begin.

When you are ready, please click "Continue" to go to the instructions.

Continue

In this experiment, you will need to allocate 100 "tokens" over two different "Options" (A and B). These Options determine the actual amount of money that you will be paid at the end of the experiment. Option A will always give you a certain payoff whereas Option B will give you either high or low payoff. In order to determine whether you receive the high or low payoff from Option B, the computer will randomly draw a ball from your urn that has 100 balls where each ball is either **GREEN** or **ORANGE**. If a **GREEN** ball is drawn you will receive the high payoff and if an **ORANGE** ball is drawn, you will receive the low payoff. In later instructions, we will describe how the number of **GREEN** and **ORANGE** balls in your urn is determined.

You will make a decision to allocate 100 tokens in many "periods" with different dollar-amount payoffs associated with each Option. Each period always has only two options: Options A and B. However, the payoffs associated with these two Options will be different between periods. This means that your earnings from a **GREEN** or **ORANGE** ball will be different between periods.

For example, consider the Options A and B below. Option A gives you $7 for certain. Option B gives you $13 if a **GREEN** ball is drawn or $5 if an **ORANGE** ball is drawn. During the experiment, this information will always be represented in two boxes in the same way that it is shown below.

Option A:	Option B:
**$7** for certain	**$13** if **GREEN** ball<br>**$5** if **ORANGE** ball

You will be given 100 tokens to distribute across these two Options. If you want, you can put all 100 tokens into Option A, which will give you $7 for certain. You can also put all 100 tokens into Option B, which will give you $13 if a **GREEN** ball is drawn or $5 if an **ORANGE** ball is drawn. The purpose of using the tokens is that, if you want, you can submit a "mixture" of the two Options as your decision.

To see how mixtures work, click "Continue".

Continue

Your earnings in each period are dependent on how many tokens you choose to allocate between the two Options. Consider Options A and B from the last screen:

Option A:	Option B:
**$7** for certain	**$13** if **GREEN** ball<br>**$5** if **ORANGE** ball

For example, suppose that you want to allocate 70 tokens into Option A and 30 tokens into Option B. These numbers are chosen purely as an example. With this allocation, you get (70/100)*$7 = $4.9 for certain because of the 70 tokens allocated to Option A. On top of that, you get an amount that depends on whether the ball is **GREEN** or **ORANGE**:

If the ball is **GREEN**, you also get (30/100)*$13 = $3.9, for a total earnings of $4.9 + $3.9 = $8.80
If the ball is **ORANGE**, you also get (30/100)*$5 = $1.5, for a total earnings of $4.9 + $1.5 = $6.40

So, your total earnings from a **GREEN** or **ORANGE** ball depends on the number of tokens you allocate to each Option. Because this calculation is a bit complicated, each period will have a "slider" tool that you can use to calculate your possible earnings of each decision. The slider tool automatically calculates your earnings if a **GREEN** or **ORANGE** ball is drawn using that allocation.

Click "Continue" to learn more about the slider.

Continue

How do I use the slider?

Using the same Options as the previous screen, click on the line to activate the slider. After the slider is activated, drag and release the yellow slider to select the fraction of tokens you want to allocate to either Option (A or B). Imagine the slider as representing the proportion of 100 tokens that you want to allocate to either Option.

You can see the amount of tokens invested into Options A and B from the updated information below the slider. The amount of tokens in each Option is represented in number form and in the height of the yellow bars. As you can see, different mixture decisions lead to different payoffs for drawing a **GREEN** or **ORANGE** ball. These payoffs represent possible total payoffs that you could earn for that period.

To do some practice examples using this slider, click "Continue".

Option A:	Option B:
**$7** for certain	**$13** if **GREEN** ball<br>**$5** if **ORANGE** ball

Please click on the line to activate the slider

Continue

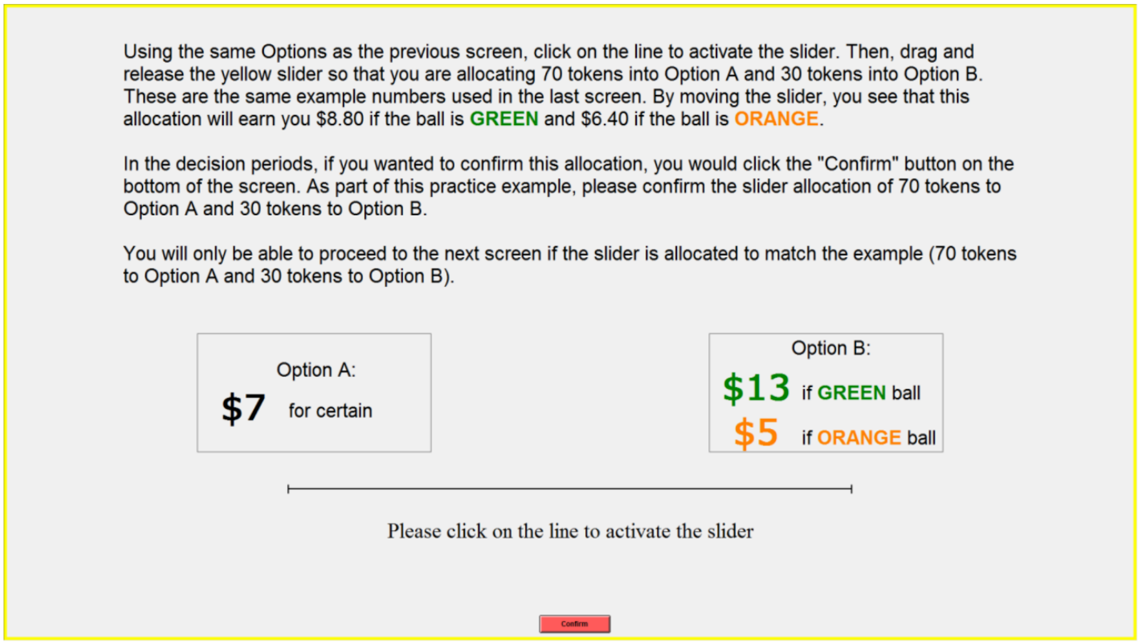
Using the same Options as the previous screen, click on the line to activate the slider. Then, drag and release the yellow slider so that you are allocating 70 tokens into Option A and 30 tokens into Option B. These are the same example numbers used in the last screen. By moving the slider, you see that this allocation will earn you $8.80 if the ball is GREEN and $6.40 if the ball is ORANGE.
In the decision periods, if you wanted to confirm this allocation, you would click the "Confirm" button on the bottom of the screen. As part of this practice example, please confirm the slider allocation of 70 tokens to Option A and 30 tokens to Option B.
You will only be able to proceed to the next screen if the slider is allocated to match the example (70 tokens to Option A and 30 tokens to Option B).
Option A:
$7 for certain
Option B:
$13 if GREEN ball
$5 if ORANGE ball
Please click on the line to activate the slider
Confirm

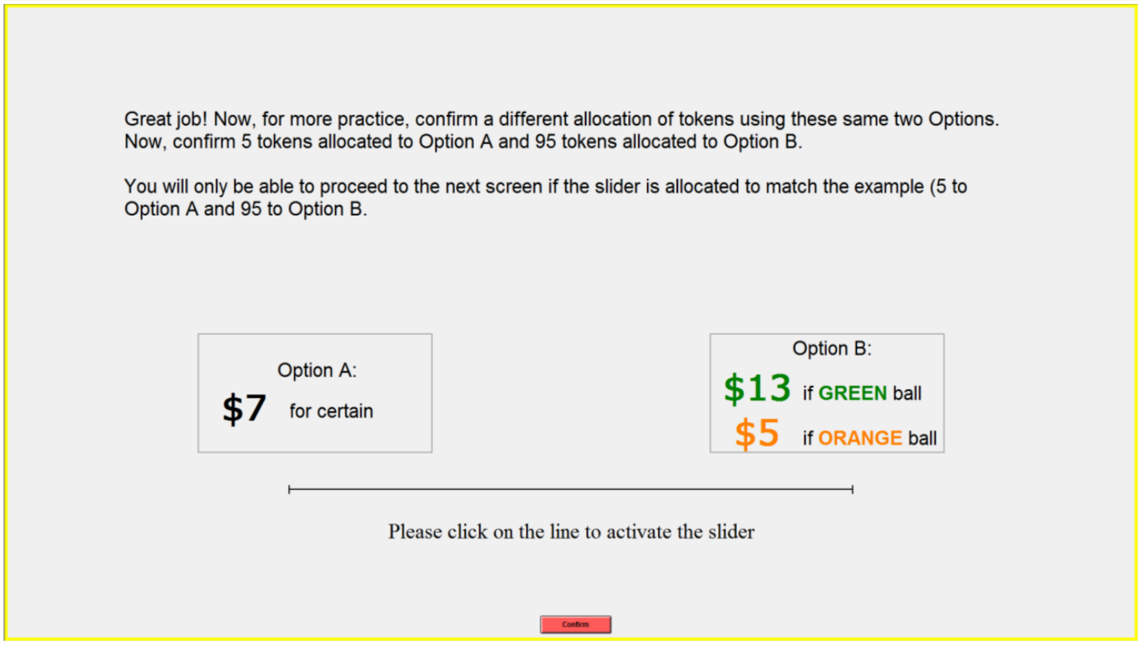
Great job! Now, for more practice, confirm a different allocation of tokens using these same two Options. Now, confirm 5 tokens allocated to Option A and 95 tokens allocated to Option B.
You will only be able to proceed to the next screen if the slider is allocated to match the example (5 to Option A and 95 to Option B.
Option A:
$7 for certain
Option B:
$13 if GREEN ball
$5 if ORANGE ball
Please click on the line to activate the slider
Confirm

Great job! Remember, the slider gives you information about your total earnings if a **GREEN** or an **ORANGE** ball is drawn. As a practice question, use the information in the slider to answer the question below.

You will only be able to proceed to the next screen if the your input is correct.

Type in your earnings when you allocate 88 tokens allocated to Option A, 12 tokens to Option B, and an **ORANGE** ball is drawn (do not use a dollar sign).

Option A:	Option B:
**$7** for certain	**$13** if **GREEN** ball<br>**$5** if **ORANGE** ball

Please click on the line to activate the slider

Confirm

*How many **GREEN** and **ORANGE** balls are in my urn?*

In the previous screens you learned how to use the slider tool to make your decision. As you noticed, your earnings depend on whether a **GREEN** or **ORANGE** ball is drawn from your urn. This fact will be the same for every period in the experiment. However, depending on the period, the number of **GREEN** and **ORANGE** balls in your urn will be determined in one of the three possible methods that are explained over the next few screens.

Method 1.) In some periods, the number of **GREEN** and **ORANGE** balls in your urn will be displayed on the screen. In these periods, your payoff will not depend on the decision of any other participant in the room. This is an example of the text you will see at the top of the screen. In this example, there are 80 **GREEN** balls and 20 **ORANGE** balls in your urn.

METHOD 1 **Balls in your urn:** **# GREEN balls: 80**
# ORANGE balls: 20

Please click "Continue" to move forward.

Continue

*How many **GREEN** and **ORANGE** balls are in my urn?*

In the previous screens you learned how to use the slider tool to make your decision. As you noticed, your earnings depends on whether a **GREEN** or **ORANGE** ball is drawn from your urn. This fact will be the same for every period in the experiment. However, depending on the period, the number of **GREEN** and **ORANGE** balls in your urn will be determined in one of the three possible methods that are explained over the next few screens.

Method 1.) In some periods, the number of **GREEN** and **ORANGE** balls in your urn will be displayed on the screen. In these periods, your payoff will not depend on the decision of any other participant in the room. This is an example of the text you will see at the top of the screen. In this example, there are 80 **GREEN** and 20 **ORANGE** balls in your urn.

METHOD 1 **Balls in your urn:** **# GREEN balls: 80**
# ORANGE balls: 20

In other periods, the total number of **GREEN** and **ORANGE** balls in your urn will not be told to you when you make your decision. Instead, you will be anonymously paired with another subject in the room and the number of **GREEN** and **ORANGE** balls will be determined by that subject's choice to allocate their 100 tokens. This pairing does not depend in any way on the decisions of any subject in any past period. So, the subject you are paired with is randomly picked by the computer and you will receive a new pairing in every period. In a period where you are paired with another subject, the **OTHER SUBJECT** will be making the same choice that you will be making. This means that they will be allocating 100 tokens over the exact same Options A and B that you will see on your screen at the same time. The **OTHER SUBJECT's** choice will affect the number of **GREEN** and **ORANGE** balls in your urn in two other methods.

Please click "Continue" to see instructions about Methods 2 and 3.

Continue

Method 2.) The number of **GREEN** balls in **YOUR** urn equals the amount of tokens that the **OTHER SUBJECT** allocates to Option A. The number of **ORANGE** balls in **YOUR** urn equals the amount of tokens the **OTHER SUBJECT** allocates to Option B. In these periods, you will see the following text at the top of the screen:

METHOD 2 **Balls in your urn:** **# GREEN balls**: # tokens **OTHER** puts into Option **A**
# ORANGE balls: # tokens **OTHER** puts into Option **B**

In these periods, your choice affects the **OTHER SUBJECT's** urn in the same way. So the number of **GREEN** balls in **THEIR** urn depends on the amount of tokens **YOU** allocate to Option A and the number of **ORANGE** balls in **THEIR** urn depends on the amount of tokens **YOU** allocate to Option B.

So, one way to think about it is the following: Every token that the **OTHER SUBJECT** allocates into Option A becomes a **GREEN** ball in your urn. Similarly, every token that you allocate into Option A becomes a **GREEN** ball in the **OTHER SUBJECT's** urn.

Please click "Continue" to learn about Method 3.

Continue

Method 2.) The number of **GREEN** balls in **YOUR** urn equals the amount of tokens that the **OTHER SUBJECT** allocates to Option A. The number of **ORANGE** balls in **YOUR** urn equals the amount of tokens the **OTHER SUBJECT** allocates to Option B. In these periods, you will see the following text at the top of the screen:

METHOD 2 **Balls in your urn:** **# GREEN balls**: # tokens **OTHER** puts into Option **A**
# ORANGE balls: # tokens **OTHER** puts into Option **B**

In these periods, your choice affects the **OTHER SUBJECT's** urn in the same way. So the number of **GREEN** balls in **THEIR** urn depends on the amount of tokens **YOU** allocate to Option A and the number of **ORANGE** balls in **THEIR** urn depends on the amount of tokens **YOU** allocate to Option B.

So, one way to think about it is the following: Every token that the **OTHER SUBJECT** allocates into Option A becomes a **GREEN** ball in your urn. Similarly, every token that you allocate into Option A becomes a **GREEN** ball in the **OTHER SUBJECT's** urn.

Method 3.) The number of **GREEN** balls in **YOUR** urn equals the amount of tokens that the **OTHER SUBJECT** allocates to Option B. The number of **ORANGE** balls in **YOUR** urn equals the amount of tokens the **OTHER SUBJECT** allocates to Option A. In these periods, you will see the following text at the top of the screen:

METHOD 3 **Balls in your urn:** **# GREEN balls**: # tokens **OTHER** puts into Option **B**
# ORANGE balls: # tokens **OTHER** puts into Option **A**

Once again, your choice affects the **OTHER SUBJECT's** urn in the same way. So the number of **GREEN** balls in **THEIR** urn depends on the amount of tokens **YOU** allocate to Option B and the number of **ORANGE** balls in **THEIR** urn depends on the amount of tokens **YOU** allocate to Option A.

So, one way to think about it is the following: Every token that the **OTHER SUBJECT** allocates into Option A becomes an **ORANGE** ball in your urn. Similarly, every token that you allocate into Option A becomes an **ORANGE** ball in the **OTHER SUBJECT's** urn.

Please click "Continue" to advance.

Continue

In summary, the number of **GREEN** balls in your urn is either displayed on the screen (Method 1), determined by the number of tokens the other allocates to Option A (Method 2), or determined by the number of tokens the other allocates to Option B (Method 3).

When you are paired with another subject in the room (Methods 2 and 3), your choice will affect the number of **GREEN** balls in their urn in the same way that their choice affects the number of **GREEN** balls in your urn. So, in other words, if you are in Method 2, then the other subject you are paired with is also in Method 2. Their screen will look identical to your screen.

You will always be informed about which Method is being used during a period. It is very important that in every period you pay attention to the Method being used to determine the amount of **GREEN** and **ORANGE** balls in your urn. In the decision periods, you will make a choice using the same Method 4 times in a row. Then the text "New Method" will flash on your screen and the Method will be changed. Then you will make a choice using this same new Method 4 times in a row. This process will continue many times until the experiment is over.

Please click "Continue" to advance to a practice quiz.

Continue

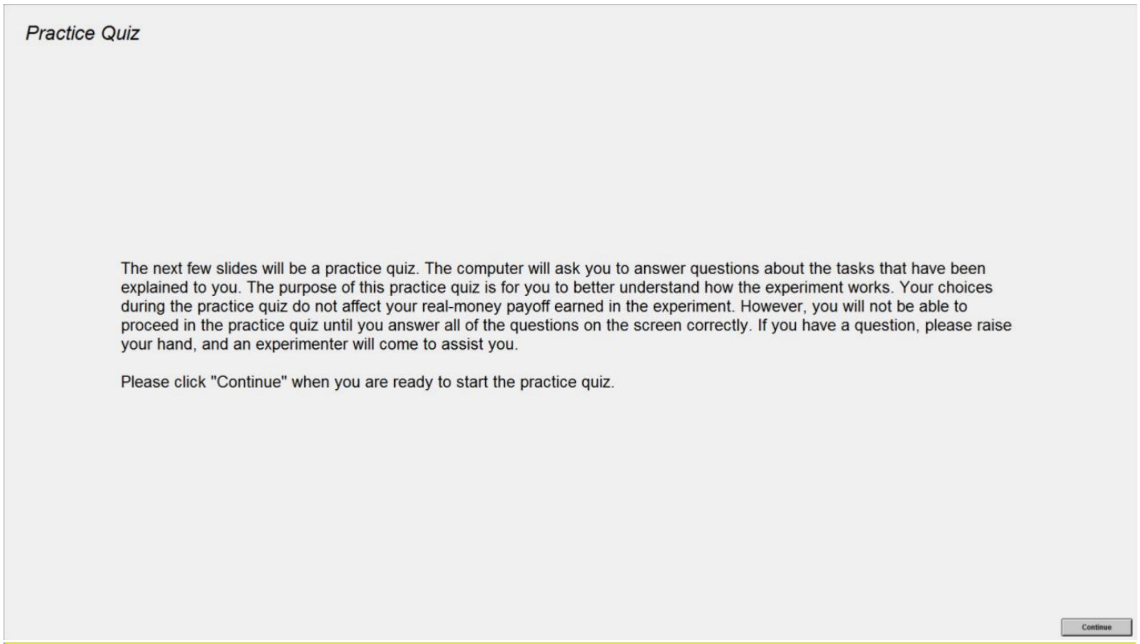
Practice Quiz
The next few slides will be a practice quiz. The computer will ask you to answer questions about the tasks that have been explained to you. The purpose of this practice quiz is for you to better understand how the experiment works. Your choices during the practice quiz do not affect your real-money payoff earned in the experiment. However, you will not be able to proceed in the practice quiz until you answer all of the questions on the screen correctly. If you have a question, please raise your hand, and an experimenter will come to assist you.
Please click "Continue" when you are ready to start the practice quiz.
Continue

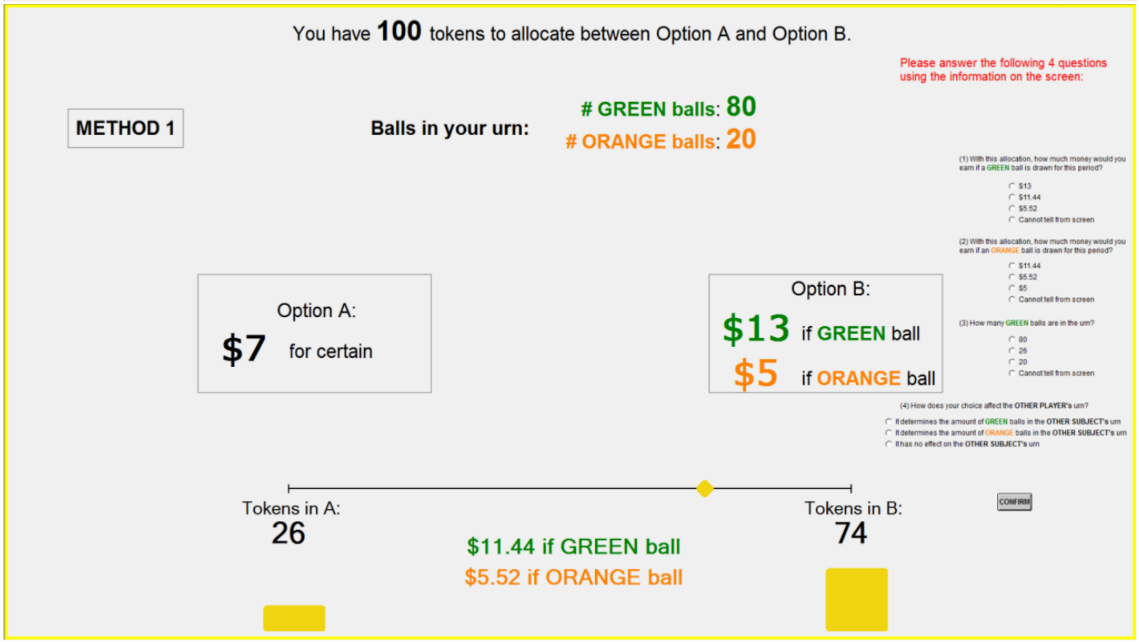
You have 100 tokens to allocate between Option A and Option B.
Please answer the following 4 questions using the information on the screen:
METHOD 1
Balls in your urn:
GREEN balls: 80
ORANGE balls: 20
(1) With this allocation, how much money would you earn if a GREEN ball is drawn for this period?
$13
$11.44
$5.52
Cannot tell from screen
(2) With this allocation, how much money would you earn if an ORANGE ball is drawn for this period?
$11.44
$5.52
$5
Cannot tell from screen
(3) How many GREEN balls are in the urn?
80
26
20
Cannot tell from screen
(4) How does your choice affect the OTHER PLAYER's urn?
It determines the amount of GREEN balls in the OTHER SUBJECT's urn
It determines the amount of ORANGE balls in the OTHER SUBJECT's urn
It has no effect on the OTHER SUBJECT's urn
Option A:
$7 for certain
Option B:
$13 if GREEN ball
$5 if ORANGE ball
Tokens in A:
26
Tokens in B:
74
CONFIRM
$11.44 if GREEN ball
$5.52 if ORANGE ball

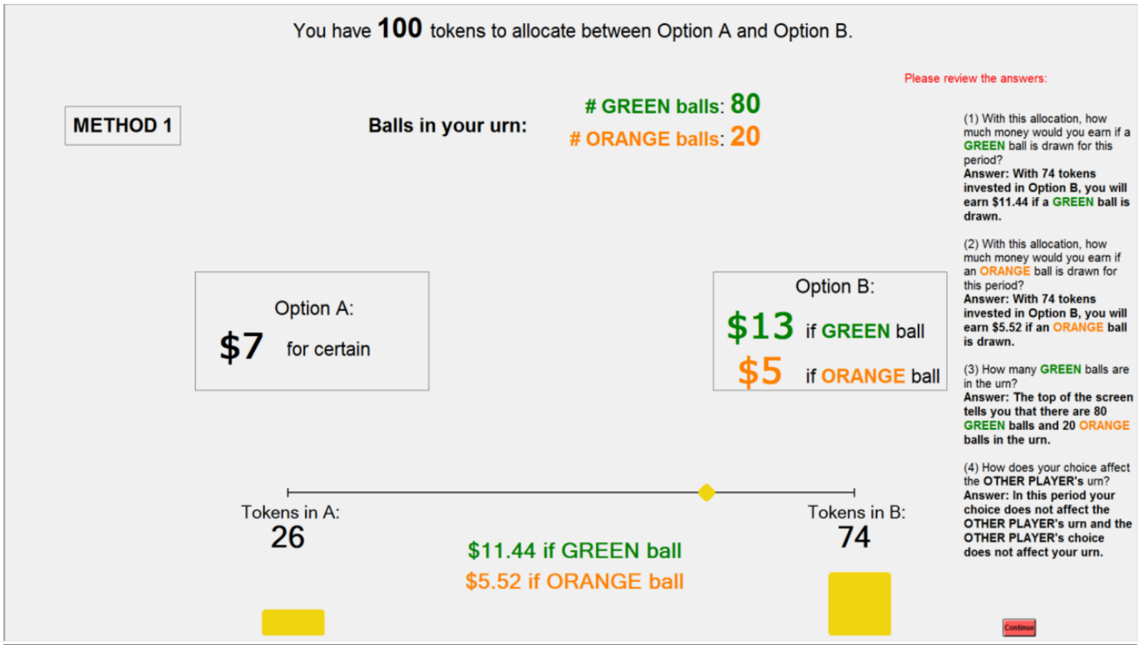
You have 100 tokens to allocate between Option A and Option B.
Please review the answers:
METHOD 1
Balls in your urn:
GREEN balls: 80
ORANGE balls: 20
(1) With this allocation, how much money would you earn if a GREEN ball is drawn for this period?
Answer: With 74 tokens invested in Option B, you will earn $11.44 if a GREEN ball is drawn.
(2) With this allocation, how much money would you earn if an ORANGE ball is drawn for this period?
Answer: With 74 tokens invested in Option B, you will earn $5.52 if an ORANGE ball is drawn.
(3) How many GREEN balls are in the urn?
Answer: The top of the screen tells you that there are 80 GREEN balls and 20 ORANGE balls in the urn.
(4) How does your choice affect the OTHER PLAYER's urn?
Answer: In this period your choice does not affect the OTHER PLAYER's urn and the OTHER PLAYER's choice does not affect your urn.
Option A:
$7 for certain
Option B:
$13 if GREEN ball
$5 if ORANGE ball
Tokens in A:
26
Tokens in B:
74
$11.44 if GREEN ball
$5.52 if ORANGE ball
Continue

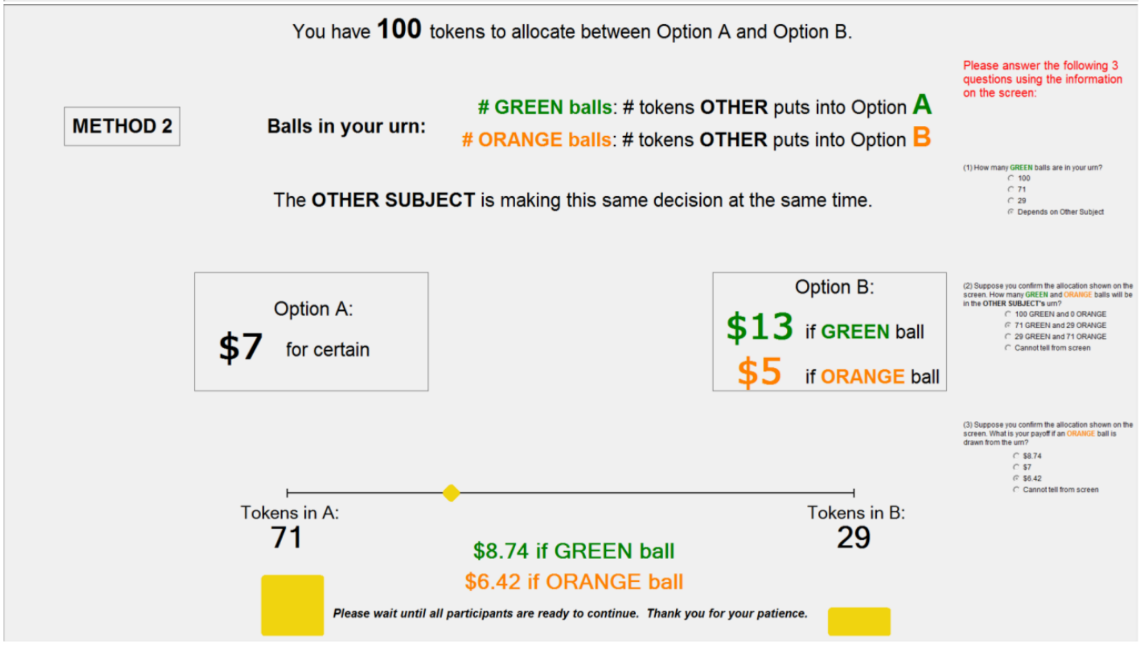
You have 100 tokens to allocate between Option A and Option B.
Please answer the following 3 questions using the information on the screen:
METHOD 2
Balls in your urn:
GREEN balls: # tokens OTHER puts into Option A
ORANGE balls: # tokens OTHER puts into Option B
The OTHER SUBJECT is making this same decision at the same time.
(1) How many GREEN balls are in your urn?
100
71
29
Depends on Other Subject
(2) Suppose you confirm the allocation shown on the screen. How many GREEN and ORANGE balls will be in the OTHER SUBJECT's urn?
100 GREEN and 0 ORANGE
71 GREEN and 29 ORANGE
29 GREEN and 71 ORANGE
Cannot tell from screen
(3) Suppose you confirm the allocation shown on the screen. What is your payoff if an ORANGE ball is drawn from the urn?
$8.74
$7
$6.42
Cannot tell from screen
Option A:
$7 for certain
Option B:
$13 if GREEN ball
$5 if ORANGE ball
Tokens in A:
71
Tokens in B:
29
$8.74 if GREEN ball
$6.42 if ORANGE ball
Please wait until all participants are ready to continue. Thank you for your patience.

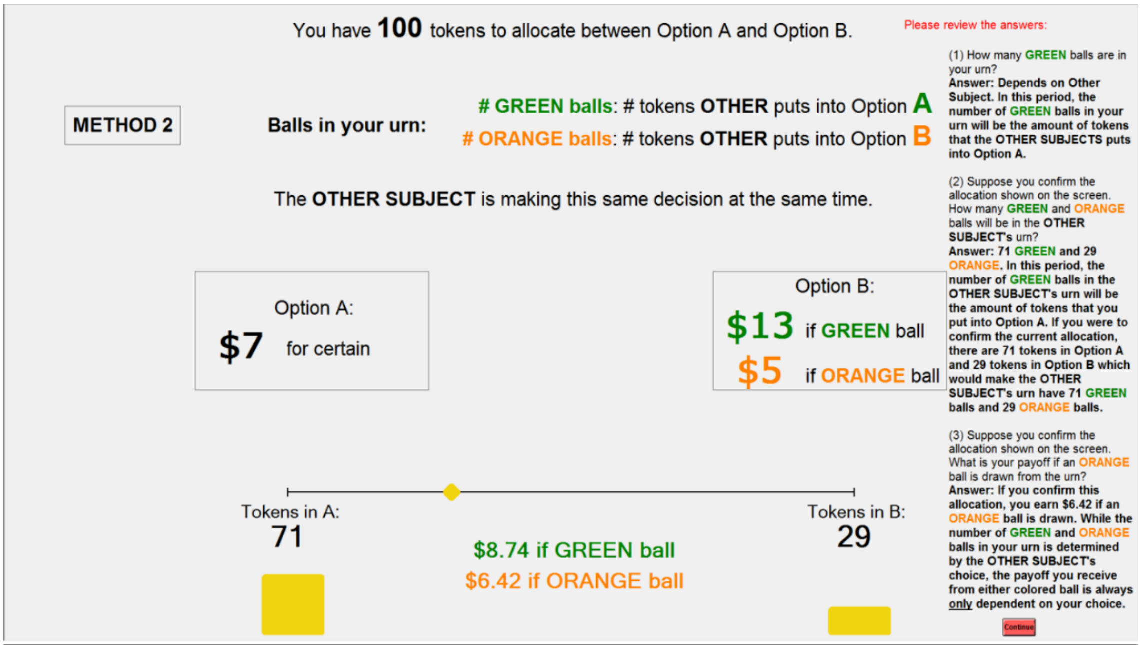
You have 100 tokens to allocate between Option A and Option B.
METHOD 2
Balls in your urn:
GREEN balls: # tokens OTHER puts into Option A
ORANGE balls: # tokens OTHER puts into Option B
The OTHER SUBJECT is making this same decision at the same time.
Option A:
$7 for certain
Option B:
$13 if GREEN ball
$5 if ORANGE ball
Tokens in A:
71
Tokens in B:
29
$8.74 if GREEN ball
$6.42 if ORANGE ball
Please review the answers:
(1) How many GREEN balls are in your urn?
Answer: Depends on Other Subject. In this period, the number of GREEN balls in your urn will be the amount of tokens that the OTHER SUBJECTS puts into Option A.
(2) Suppose you confirm the allocation shown on the screen. How many GREEN and ORANGE balls will be in the OTHER SUBJECT's urn?
Answer: 71 GREEN and 29 ORANGE. In this period, the number of GREEN balls in the OTHER SUBJECT's urn will be the amount of tokens that you put into Option A. If you were to confirm the current allocation, there are 71 tokens in Option A and 29 tokens in Option B which would make the OTHER SUBJECT's urn have 71 GREEN balls and 29 ORANGE balls.
(3) Suppose you confirm the allocation shown on the screen. What is your payoff if an ORANGE ball is drawn from the urn?
Answer: If you confirm this allocation, you earn $6.42 if an ORANGE ball is drawn. While the number of GREEN and ORANGE balls in your urn is determined by the OTHER SUBJECT's choice, the payoff you receive from either colored ball is always only dependent on your choice.
Continue

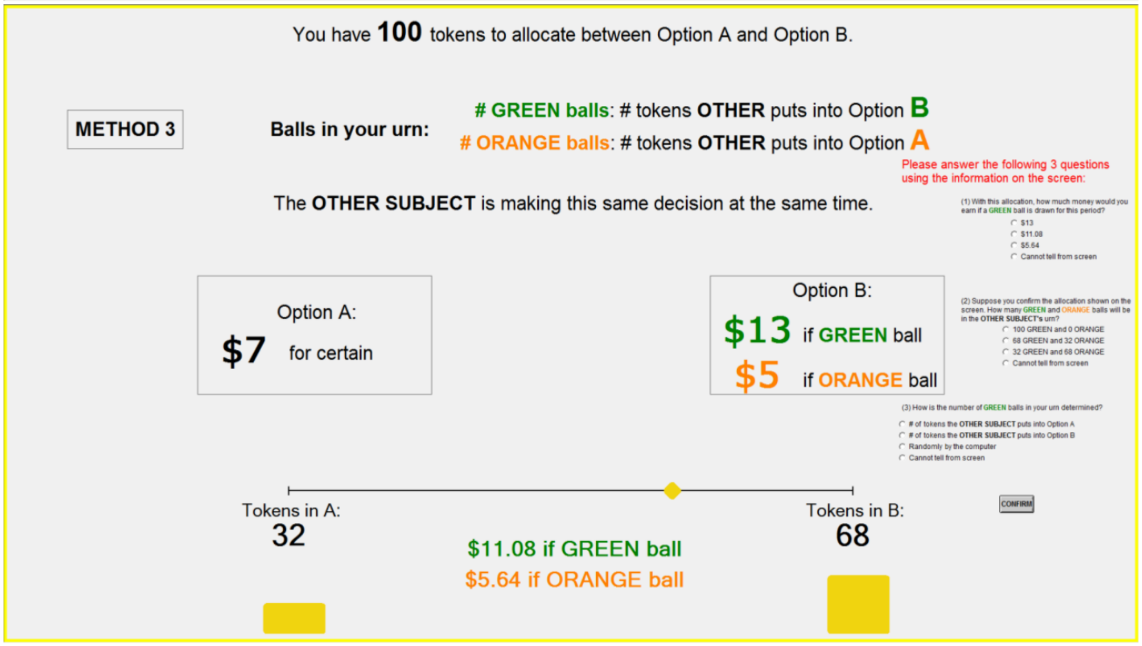
You have 100 tokens to allocate between Option A and Option B.
METHOD 3
Balls in your urn:
GREEN balls: # tokens OTHER puts into Option B
ORANGE balls: # tokens OTHER puts into Option A
The OTHER SUBJECT is making this same decision at the same time.
Option A:
$7 for certain
Option B:
$13 if GREEN ball
$5 if ORANGE ball
Tokens in A:
32
Tokens in B:
68
$11.08 if GREEN ball
$5.64 if ORANGE ball
Please answer the following 3 questions using the information on the screen:
(1) With this allocation, how much money would you earn if a GREEN ball is drawn for this period?
$13
$11.08
$5.64
Cannot tell from screen
(2) Suppose you confirm the allocation shown on the screen. How many GREEN and ORANGE balls will be in the OTHER SUBJECT's urn?
100 GREEN and 0 ORANGE
68 GREEN and 32 ORANGE
32 GREEN and 68 ORANGE
Cannot tell from screen
(3) How is the number of GREEN balls in your urn determined?
# of tokens the OTHER SUBJECT puts into Option A
# of tokens the OTHER SUBJECT puts into Option B
Randomly by the computer
Cannot tell from screen
CONFIRM

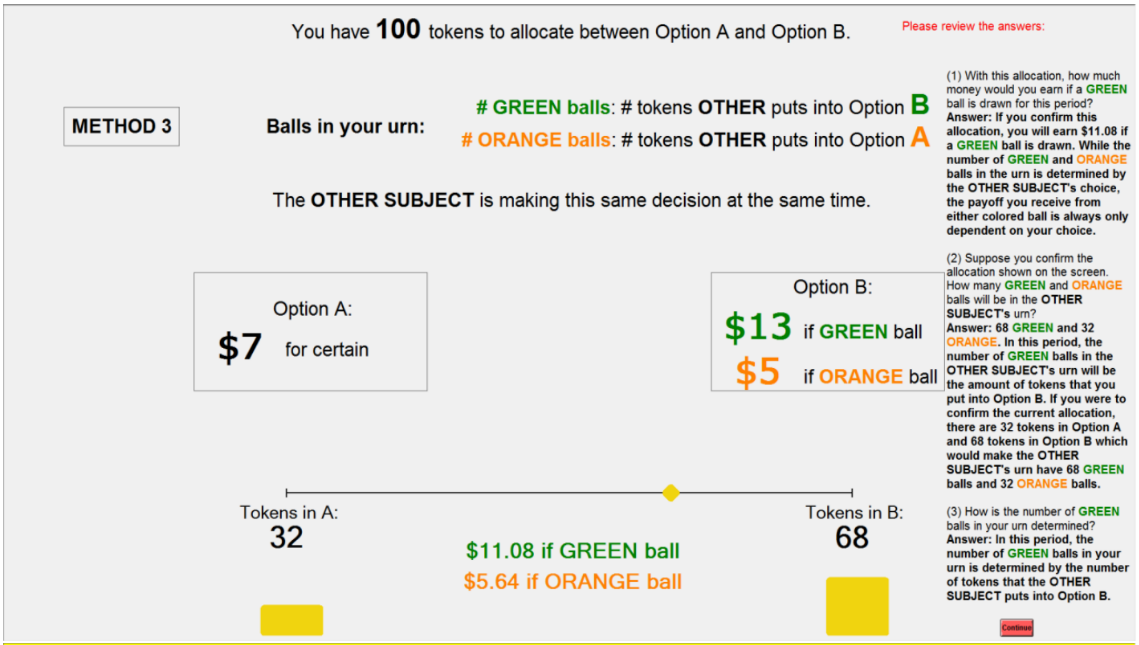
You have 100 tokens to allocate between Option A and Option B.
Please review the answers:
METHOD 3
Balls in your urn:
GREEN balls: # tokens OTHER puts into Option B
ORANGE balls: # tokens OTHER puts into Option A
The OTHER SUBJECT is making this same decision at the same time.
Option A:
$7 for certain
Option B:
$13 if GREEN ball
$5 if ORANGE ball
Tokens in A:
32
Tokens in B:
68
$11.08 if GREEN ball
$5.64 if ORANGE ball
(1) With this allocation, how much money would you earn if a GREEN ball is drawn for this period?
Answer: If you confirm this allocation, you will earn $11.08 if a GREEN ball is drawn. While the number of GREEN and ORANGE balls in the urn is determined by the OTHER SUBJECT's choice, the payoff you receive from either colored ball is always only dependent on your choice.
(2) Suppose you confirm the allocation shown on the screen. How many GREEN and ORANGE balls will be in the OTHER SUBJECT's urn?
Answer: 68 GREEN and 32 ORANGE. In this period, the number of GREEN balls in the OTHER SUBJECT's urn will be the amount of tokens that you put into Option B. If you were to confirm the current allocation, there are 32 tokens in Option A and 68 tokens in Option B which would make the OTHER SUBJECT's urn have 68 GREEN balls and 32 ORANGE balls.
(3) How is the number of GREEN balls in your urn determined?
Answer: In this period, the number of GREEN balls in your urn is determined by the number of tokens that the OTHER SUBJECT puts into Option B.
Continue

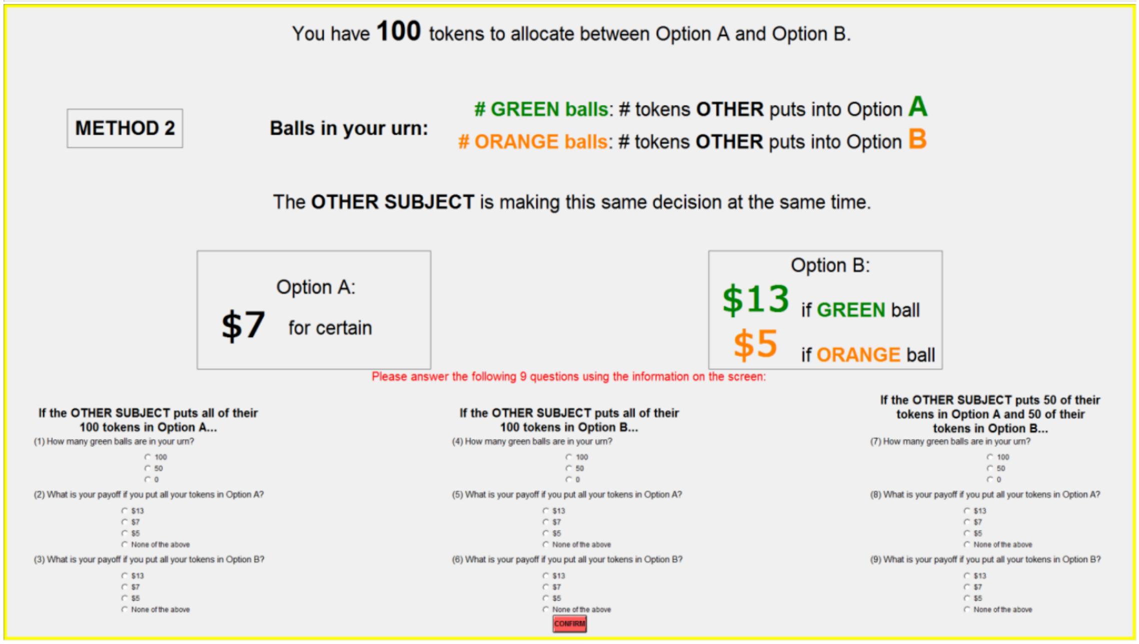
You have 100 tokens to allocate between Option A and Option B.
METHOD 2
Balls in your urn:
GREEN balls: # tokens OTHER puts into Option A
ORANGE balls: # tokens OTHER puts into Option B
The OTHER SUBJECT is making this same decision at the same time.
Option A:
$7 for certain
Option B:
$13 if GREEN ball
$5 if ORANGE ball
Please answer the following 9 questions using the information on the screen:
If the OTHER SUBJECT puts all of their 100 tokens in Option A...
(1) How many green balls are in your urn?
100
50
0
(2) What is your payoff if you put all your tokens in Option A?
$13
$7
$5
None of the above
(3) What is your payoff if you put all your tokens in Option B?
$13
$7
$5
None of the above
If the OTHER SUBJECT puts all of their 100 tokens in Option B...
(4) How many green balls are in your urn?
100
50
0
(5) What is your payoff if you put all your tokens in Option A?
$13
$7
$5
None of the above
(6) What is your payoff if you put all your tokens in Option B?
$13
$7
$5
None of the above
CONFIRM
If the OTHER SUBJECT puts 50 of their tokens in Option A and 50 of their tokens in Option B...
(7) How many green balls are in your urn?
100
50
0
(8) What is your payoff if you put all your tokens in Option A?
$13
$7
$5
None of the above
(9) What is your payoff if you put all your tokens in Option B?
$13
$7
$5
None of the above

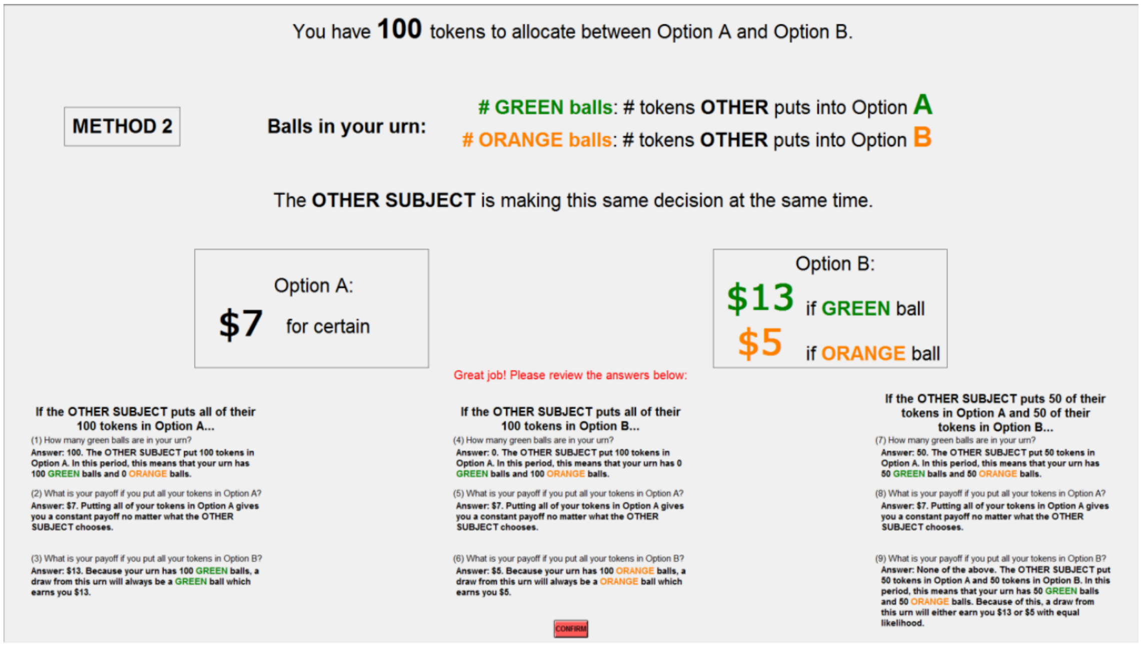
You have 100 tokens to allocate between Option A and Option B.
METHOD 2
Balls in your urn:
GREEN balls: # tokens OTHER puts into Option A
ORANGE balls: # tokens OTHER puts into Option B
The OTHER SUBJECT is making this same decision at the same time.
Option A:
$7 for certain
Option B:
$13 if GREEN ball
$5 if ORANGE ball
Great job! Please review the answers below:
If the OTHER SUBJECT puts all of their 100 tokens in Option A...
(1) How many green balls are in your urn?
Answer: 100. The OTHER SUBJECT put 100 tokens in Option A. In this period, this means that your urn has 100 GREEN balls and 0 ORANGE balls.
(2) What is your payoff if you put all your tokens in Option A?
Answer: $7. Putting all of your tokens in Option A gives you a constant payoff no matter what the OTHER SUBJECT chooses.
(3) What is your payoff if you put all your tokens in Option B?
Answer: $13. Because your urn has 100 GREEN balls, a draw from this urn will always be a GREEN ball which earns you $13.
If the OTHER SUBJECT puts all of their 100 tokens in Option B...
(4) How many green balls are in your urn?
Answer: 0. The OTHER SUBJECT put 100 tokens in Option A. In this period, this means that your urn has 0 GREEN balls and 100 ORANGE balls.
(5) What is your payoff if you put all your tokens in Option A?
Answer: $7. Putting all of your tokens in Option A gives you a constant payoff no matter what the OTHER SUBJECT chooses.
(6) What is your payoff if you put all your tokens in Option B?
Answer: $5. Because your urn has 100 ORANGE balls, a draw from this urn will always be a ORANGE ball which earns you $5.
If the OTHER SUBJECT puts 50 of their tokens in Option A and 50 of their tokens in Option B...
(7) How many green balls are in your urn?
Answer: 50. The OTHER SUBJECT put 50 tokens in Option A. In this period, this means that your urn has 50 GREEN balls and 50 ORANGE balls.
(8) What is your payoff if you put all your tokens in Option A?
Answer: $7. Putting all of your tokens in Option A gives you a constant payoff no matter what the OTHER SUBJECT chooses.
(9) What is your payoff if you put all your tokens in Option B?
Answer: None of the above. The OTHER SUBJECT put 50 tokens in Option A and 50 tokens in Option B. In this period, this means that your urn has 50 GREEN balls and 50 ORANGE balls. Because of this, a draw from this urn will either earn you $13 or $5 with equal likelihood.
CONFIRM

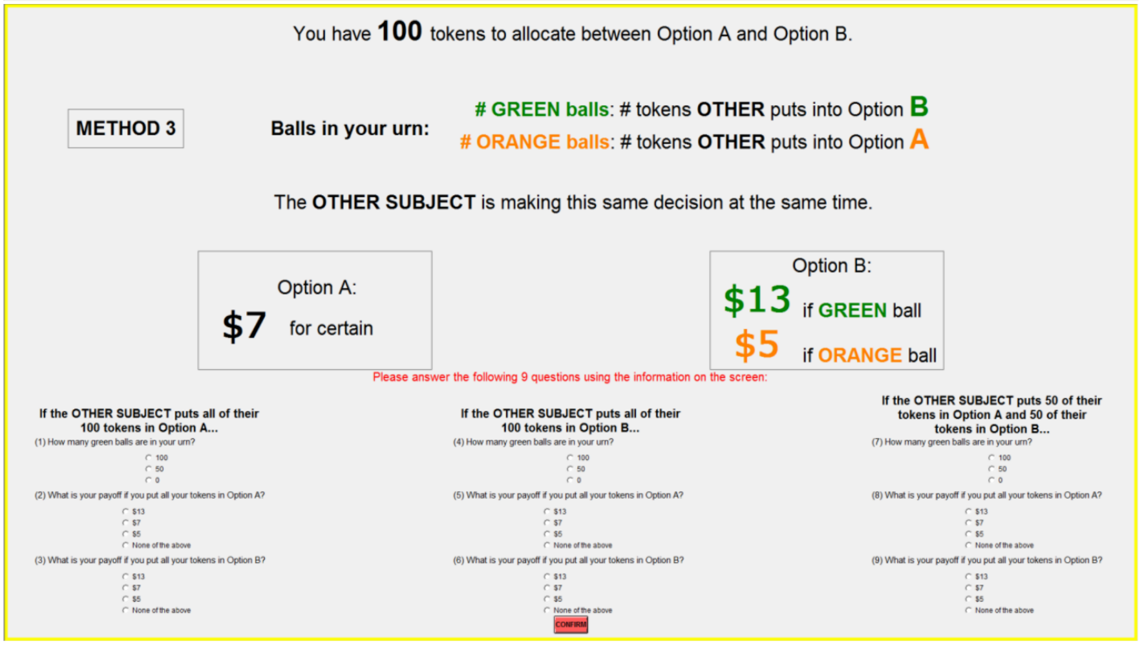
You have 100 tokens to allocate between Option A and Option B.
METHOD 3
Balls in your urn:
GREEN balls: # tokens OTHER puts into Option B
ORANGE balls: # tokens OTHER puts into Option A
The OTHER SUBJECT is making this same decision at the same time.
Option A:
$7 for certain
Option B:
$13 if GREEN ball
$5 if ORANGE ball
Please answer the following 9 questions using the information on the screen:
If the OTHER SUBJECT puts all of their 100 tokens in Option A...
(1) How many green balls are in your urn?
100
50
0
(2) What is your payoff if you put all your tokens in Option A?
$13
$7
$5
None of the above
(3) What is your payoff if you put all your tokens in Option B?
$13
$7
$5
None of the above
If the OTHER SUBJECT puts all of their 100 tokens in Option B...
(4) How many green balls are in your urn?
100
50
0
(5) What is your payoff if you put all your tokens in Option A?
$13
$7
$5
None of the above
(6) What is your payoff if you put all your tokens in Option B?
$13
$7
$5
None of the above
If the OTHER SUBJECT puts 50 of their tokens in Option A and 50 of their tokens in Option B...
(7) How many green balls are in your urn?
100
50
0
(8) What is your payoff if you put all your tokens in Option A?
$13
$7
$5
None of the above
(9) What is your payoff if you put all your tokens in Option B?
$13
$7
$5
None of the above
CONFIRM

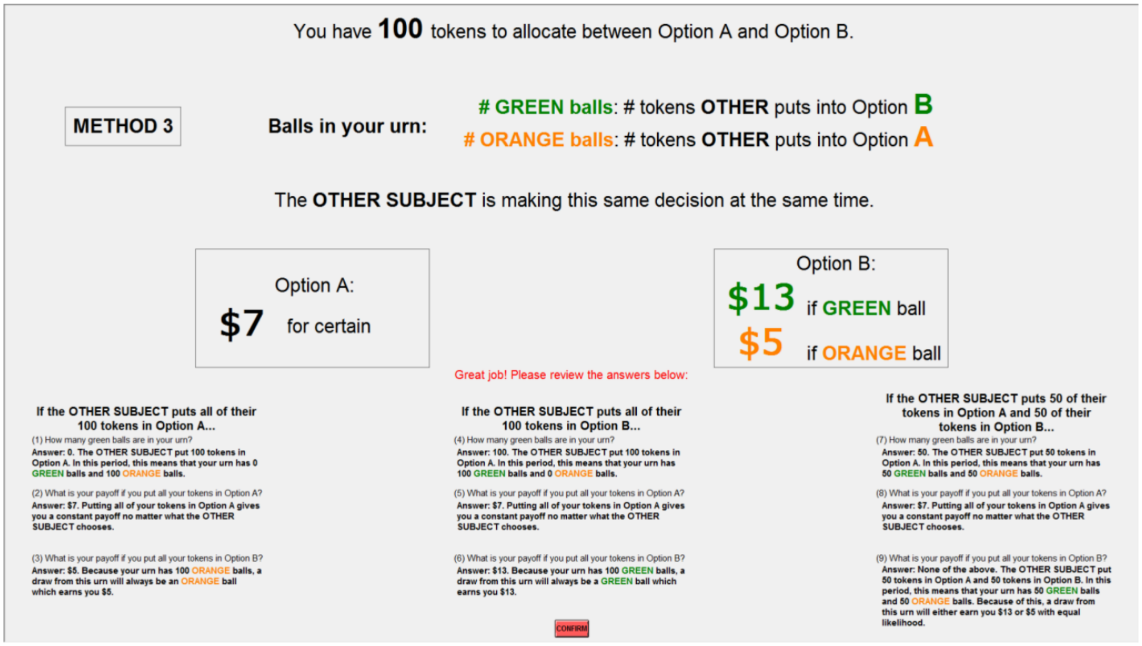

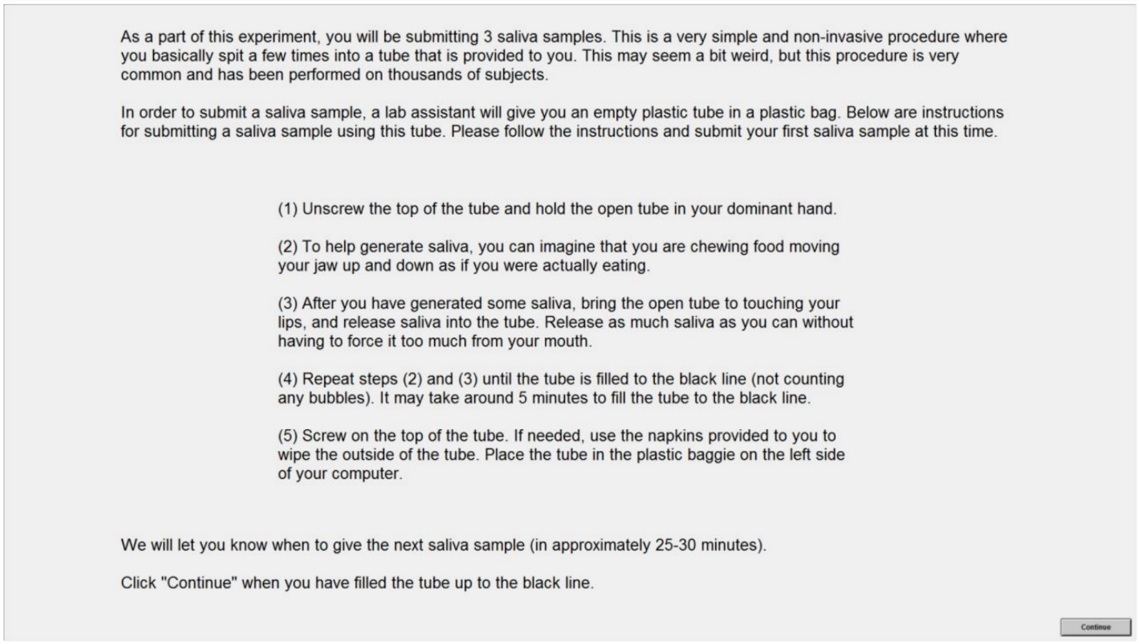

[Treatment subjects only]

For this part of the experiment, you will need to place your left hand in a bucket of ice water with your fingers spread open. It is very important that you leave your hand in the water as long as you possibly can. There is a required minimum amount of time that you need to keep your hand in the water in order to continue with the experiment. **If you do not keep your hand in the water for a long enough time to satisfy the minimum requirement, you will be asked to leave the experiment with only your $5 show-up fee.** You only have one opportunity to keep your hand in the water for a long enough time to pass the minimum threshold.

While you do not know the minimum required time, if you keep your hand in the water for 3 minutes you are guaranteed to pass this portion of the experiment and will be allowed to continue. I will announce when 3 minutes have passed. If, for some reason, you need to remove your hand before the 3 minutes are complete, please wait silently until the 3 minutes are up.

Also, we need to analyze and videotape your facial expressions during this task. It is important that you look directly into the camera for the entire time your hand is in the water.

Please remove all jewelry on your left hand and arm. The lab assistants are setting up a bucket of ice water for each of you and will be placing an elastic band on your left wrist above your wrist bone. Keeping your hand in the water means keeping the elastic band submerged underneath the water. Your time stops if your hand comes out of the water enough so that the elastic band is no longer under the water. The lab assistants will be watching the elastic band along with your video recording. Also, your fingers need to remain spread during the whole time your hand is in the water.

When everyone is ready to proceed, the experimenter will instruct everyone to begin. When the experimenter says "Begin", put your hand in the bucket of ice water and look directly into the camera. At this point, the timer will be started, and you will be told when 3 minutes have passed. Your time stops if you close your hand into a fist or if you remove your hand from the water such that the elastic band is no longer submerged.

If you feel it is necessary, please adjust your seat by using the lever on the right side of your chair.

Continue

Final Instructions

The cameras are turned off and you will not be videotaped at any other time during the experiment.

Now we are ready to begin the decision periods that will determine your dollar-amount earnings. After you have completed ALL of the periods, you will be shown a list displaying the choices that you made in every period. From this list, the computer will randomly select ONE PERIOD. Using the choices made in this period, the computer will then randomly draw ONE BALL from that period's urn. The earnings from this draw in this period will determine what you receive in the experiment (on top of the $5 show-up fee).

Because you will be paid based on the outcome of one of the upcoming periods, it is important to take your time in each period and make a choice that you are satisfied with.

Each period is unique. This means that you will never be shown the same choice twice.

If you have questions at any time during the experiment please raise your hand and an experimenter will come to assist you.
Please click "Continue" to start the decision periods.

Continue
